# Risk of second primary cancer among breast cancer patients: A systematic review and meta-analysis

**DOI:** 10.3389/fonc.2022.1094136

**Published:** 2023-01-17

**Authors:** Parynaz Parhizgar, Ayad Bahadori Monfared, Maryam Mohseny, Aliasghar Keramatinia, Seyed Saeed Hashemi Nazari, Syed Azizur Rahman, Amina Al Marzouqi, Nabeel Al-Yateem, Alireza Mosavi Jarrahi

**Affiliations:** ^1^ School of Medicine, Shahid Beheshti University of Medical Sciences, Tehran, Iran; ^2^ Department of Social Medicine, School of Medicine, Shahid Beheshti University of Medical Sciences, Tehran, Iran; ^3^ Prevention of Cardiovascular Disease Research Center, Department of Epidemiology, School of Public Health and Safety, Shahid Beheshti University of Medical Sciences, Tehran, Iran; ^4^ Department of Health Service Administration, College of Health Sciences, University of Sharjah, Sharjah, United Arab Emirates; ^5^ Department of Nursing, College of Health Sciences, University of Sharjah, Sharjah, United Arab Emirates; ^6^ Cancer and Epidemiology Research Center, West Asia Organization for Cancer Prevention, Sabzevar, Iran

**Keywords:** breast cancer, multiple primary cancer, second primary cancer, incidence, risk

## Abstract

**Objectives:**

The aim of this study was to estimate the extra risk of second primary cancer among breast cancer patients.

**Methods and materials:**

This is a systematic review. A comprehensive search of literature was performed in PubMed, Web of Science, Cochrane library, and Scopus. The search included all published studies up to October 2022. This systematic review included studies published in the English language that reported the risk of second primary non-breast cancer [*i.e.*, standardized incidence ratio (SIR)] among breast cancer patients older than 15 years. After evaluating the methodological quality of the selected studies, SIRs were pooled with consideration of heterogeneity among studies. The estimates were pooled by age and time since the diagnosis of primary breast cancer for both sexes (male and female). Age was categorized based on before 50 years and after 50 years, and time was categorized as duration of less than and more than 10 years, respectively.

**Results:**

From 2,484 articles, 30 articles were eligible for inclusion in the systematic review and meta-analysis. The studies varied in terms of population, number of cases, study design, setting, and year of implementation of the research. The estimated SIR for men and women was 1.28 (95% CI: 1.18, 1.38) and 1.27 (95% CI: 1.15, 1.39), respectively. Women diagnosed with breast cancer before menopause [SIR: 1.52 (95% CI: 1.34, 1.71) *vs*. 1.21 (95% CI: 1.08, 1.34)] as well as women after 10 years since their breast cancer diagnosis [1.33 (95% CI: 1.22, 1.431) *vs*. 1.24 (95% CI: 1.10, 1.37)] were at a higher risk of developing second primary cancer. Among men, while there were no differences in risk based on age, with the increase of time, the risk of second primary cancer was reduced [SIR: 1.22 (95% CI: 1.12, 1.33) *vs*. 1.00 (95% CI: 0.79, 1.22)].

**Conclusion:**

There is an extra risk of second primary cancer among breast cancer patients. The extra risk should be considered for further screening and preventive measures among this population.

**Systematic review registration:**

https://www.crd.york.ac.uk/prospero/display_record.php?RecordID=336062, identifier (CRD42022336062).

## Introduction

Breast cancer is one of the major health problems in the world and ranks number one in mortality among women of older age in many developed and even developing countries (1). Longer survival does not only result in a longer low-quality-of-life among the population of patients but also provides ample opportunity for the occurrence of a second primary malignancy, which somehow is related to the constitutional susceptibility of these patients to malignancy (2). According to Globocan 2020, breast cancer is one of the most common cancers in the world, with age-standardized incidence rates of up to 47.8 per 100,000 and age-standardized mortality rates of up to 13.6 per 100,000 (3). In the recent three decades, better access to health services, breast cancer screening (4), early detection (5), and advances in breast cancer treatment (hormone therapy, surgery, radiotherapy, and chemotherapy) have led to an increase in survival and a reduction in breast cancer deaths (especially in developed countries) (6–8). As patient survival increases, the risk of second primary cancers (also known as second cancers) increases and becomes a burden for patients and healthcare providers. An unexpectedly higher rate of second primary cancer among survivors of breast cancer shed light on an underlying susceptibility among this group of patients (9). The risk of second primary cancer (excluding contralateral breast cancer) has been reported to be as high as 20%–30% among breast cancer survivors (10). While all kinds of cancers are reported as second primary, cancers of the endometrium, ovary, thyroid, lung, soft tissue sarcomas, leukemia, melanoma, stomach, and colon have been reported more frequently (11, 12). The descriptive epidemiology of second primary cancer among survivors at 5, 10, and 15 years is reported to be 3.6%, 8.2%, and 13.9%, respectively (13), and patients less than 50 years of age (also premenopausal ages) are at a higher risk of developing second cancer (10, 12). Several population-based cancer registry studies have assessed the risk of second primary cancers among women diagnosed with primary breast cancer compared with the general population (10, 12, 14–18). Some studies have also examined the risk of developing second cancer based on the type of treatment (19–24), age difference (25–27), and family history (27–29). However, the risk estimates provided by these studies vary widely, and there is no consensus among the studies (in terms of the definition of first and second primary cancers and the inclusion and exclusion criteria). Studies have used different definitions for second primary cancer and different coding systems [such as International Association of Cancer Registries (IARC) and Surveillance, Epidemiology, and End Results (SEER)]. In a study by Molina et al. (7), which covered studies published up to 2013 based on the IARC definition of a second primary cancer, the standardized incidence ratio (SIR) of developing second cancer (excluding second breast cancer) was 1.17 (17% higher than expected). However, this study did not report the risk of each cancer separately. Knowing the risk of a second primary cancer in any of the organs can help policymakers prioritize resources as well as generate solid evidence on the risk of a second primary cancer among breast cancer survivors and will fill the existing gap for administrative purposes. This systematic review aims to investigate the risk and incidence of second primary non-breast malignancies among breast cancer patients (all cancers in men and women) using observational studies.

## Methods and materials

This systematic review and meta-analysis was developed based on the MOOSE Checklist for Meta-analyses of Observational Studies (30). A comprehensive search of literature was performed in PubMed, Web of Science, Cochrane library, and Scopus. The search included all published studies up to October 2022.

### Search strategy

A systematic search was carried out up to October 7, 2022. Initially, relevant keywords were identified using the primary search in PubMed/Medline; then, the search strategy was adapted accordingly for Scopus, Cochrane Library, and Web of Science. Language restrictions were considered in the search process (only English articles). The following selected keywords were used and combined in the selected search databases: multiple primary neoplasm(s), multiple primary cancer, multiple primary malignancies, second cancer, second malignancies, breast cancer, risk, and population-based. The details of the comprehensive search strategy for this systematic review are provided in the supplementary materials. Studies were entered into EndNote X9 software (Thomson Reuters, New York, NY, USA), and duplicate items were detected and removed by this software.

### Eligibility criteria

This systematic review included all studies published in the English language that reported the risk of a second primary cancer (*i*.*e*., SIR) among breast cancer patients older than 15 years. The second primary cancer was defined based on IARC definition (31) which referred to second primary cancer as more than one cancer diagnosed in the same person which is not dependent on time and not categorized as a recurrence or metastasis; in addition, only one tumor should be present in one organ or paired organ [there are two exceptions (1): Kaposi sarcoma and tumors of the hematopoietic system are considered as one tumor and (2) if tumors with a different morphology are reported in a paired organ, they are counted as two tumors].

### Exclusion criteria

Studies that used definitions other than the IARC definition for reporting second primary malignancies (such as those defined by SEER) were excluded. Articles that did not specify the coding definition were excluded if it was apparent that the definition for accepting a second primary cancer did not agree with the IARC definition. Articles reporting a second primary breast cancer usually lack information on reporting second cancer in the same or in the contralateral breast as well as histological reports of tumors. Therefore, all studies which included second primary breast cancer were excluded unless risk estimates for other sites of second primary cancer were reported. Due to the multimodal treatment of breast cancer, studies that examined the risk of second cancer based on treatment type were excluded.

### Study selection

In the selection stage, the two reviewers separately checked the title, abstract, and full text of the articles using the inclusion and exclusion criteria, and any discrepancies between the results were resolved by discussion. The reference lists of the included studies were reviewed to identify other potentially relevant studies. If more than one article were published for one population, the newer or the one with a larger population was included. The data was extracted manually by two independent researchers. Disagreements were resolved through discussion and consensus. If there were deficiencies in the required information of the selected studies, the corresponding author of the article was contacted by email, and additional information about the results of the study was requested. The following information was extracted: first authors, publication date, country of origin, study design, study follow-up period, cases (number of first and second primary cancer), mean age at cancer diagnosis (both first and second primary cancer), gender, definition of second primary cancer, inclusion criteria for their population, and estimated SIR and its associated 95% confidence intervals (CI). The following cancer sites were included in the analysis: skin (melanoma and non-melanoma), head and neck (oral cavity, pharynx, and larynx), esophagus, stomach, colorectal, liver and gallbladder and biliary tract, pancreas, lung and mediastinum, Kaposi sarcoma, leukemia, lymphoma, multiple myeloma, soft tissue sarcoma, bone, prostate, testis, kidney, bladder and urinary tract, brain and nervous system, thyroid, eye, corpus uteri, cervix uteri, ovary, adrenal gland, and parathyroid.

### Quantitative analysis

The standard incidence ratio was defined as the incidence of cancer cases in a population over a given period to the incidence of cancer that would be expected over the same period if the study population had the same age-specific rates as the standard population. If the ratio is greater than 1, it is interpreted as extra incidence in the study population. First, we calculated the standard error (SE) for each estimate (SE = √observed/expected^2^) (32). Then, we calculated pooled risk ratios (SIR) using SIR and SE. Age at primary breast cancer diagnosis was categorized as <50 years and ≥50 years, and the diagnosis of primary breast cancer to the diagnosis of second cancer was categorized as <10 years and ≥10 years. The variations in the categorization of age or time since diagnosis were standardized by pooling the categories to their nearest cutoff point to meet our study’s categorization scheme. Moreover, if the cutoff points of a study overlapped with both cutoff categories of our study, we equally divided the observed and expected values into two cutoff boundaries.

To evaluate the risk of bias in individual studies, we used the SIGN50 Scottish Intercollegiate Network checklist (33). This checklist assesses methodological quality, overall quality (based on design, internal validity, consistency, and accuracy of results), and level of evidence and finally classifies studies into three (low, acceptable, and high) categories. Using the Q-statistic and *I* ^2^ values, a heterogeneity test was carried out among the included studies. If the *I* ^2^ value is less than 50%, it was considered as without significant heterogeneity, and we pooled the estimates using the fixed-effect model. An *I* ^2^ value higher than 50% was considered as obvious heterogeneity, and we pooled estimates using the random-effect model. In addition, subgroup and sensitivity analyses were performed to identify the source of heterogeneity. The effect of a study’s quality on the estimates was measured using meta-regression and subgroup analyses. Publication bias was investigated using funnel plots (34), and Egger’s (35) test.

## Results

### Results of comprehensive search

**Figure 1:**
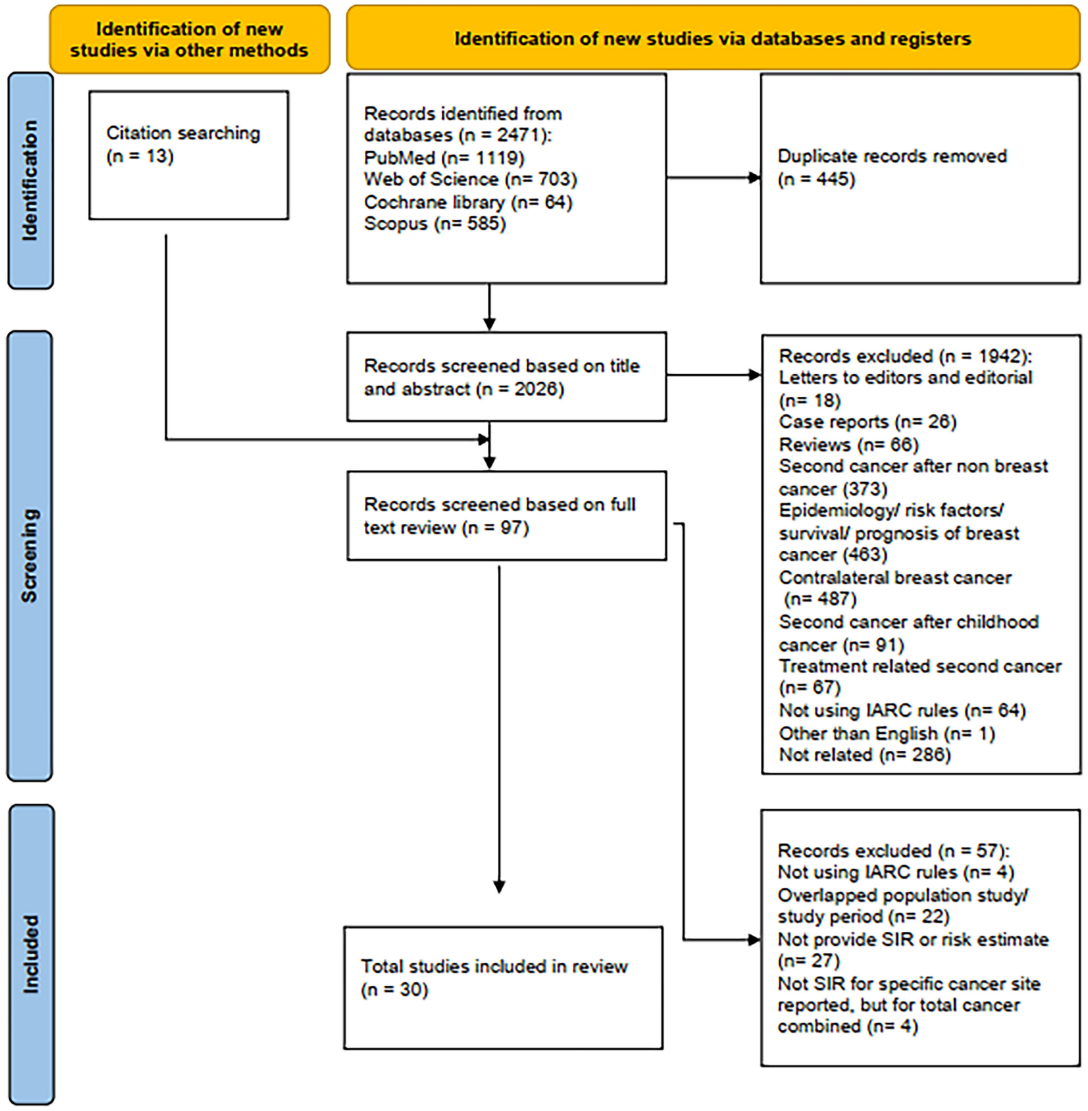
PRISMA flow chart showing the study’s search and screening procedure.

As shown in **Figure 1**, the defined search criteria in selected databases retrieved 2,471 potentially relevant articles. Due to duplication, 445 articles were removed. We screened the articles based on the title and the abstract, and 1,942 articles were excluded for different reasons. Furthermore, through a manual search of reference lists, we identified 13 articles. Therefore, 97 articles were left for full-text review, of which 57 articles were excluded: four studies did not use IARC definition (*i*.*e*., SEER, *etc.*), 27 studies did not provide SIR or risk estimate, and four studies did not provide SIR for specific cancer site but reported for total cancer combined. Population or period overlaps were also seen in 22 studies (36–57). Finally, 30 studies (10, 12, 14–17, 27, 28, 58–79) were included in the present systematic review and meta-analysis.

### Description of the included studies

**Figure 2:**
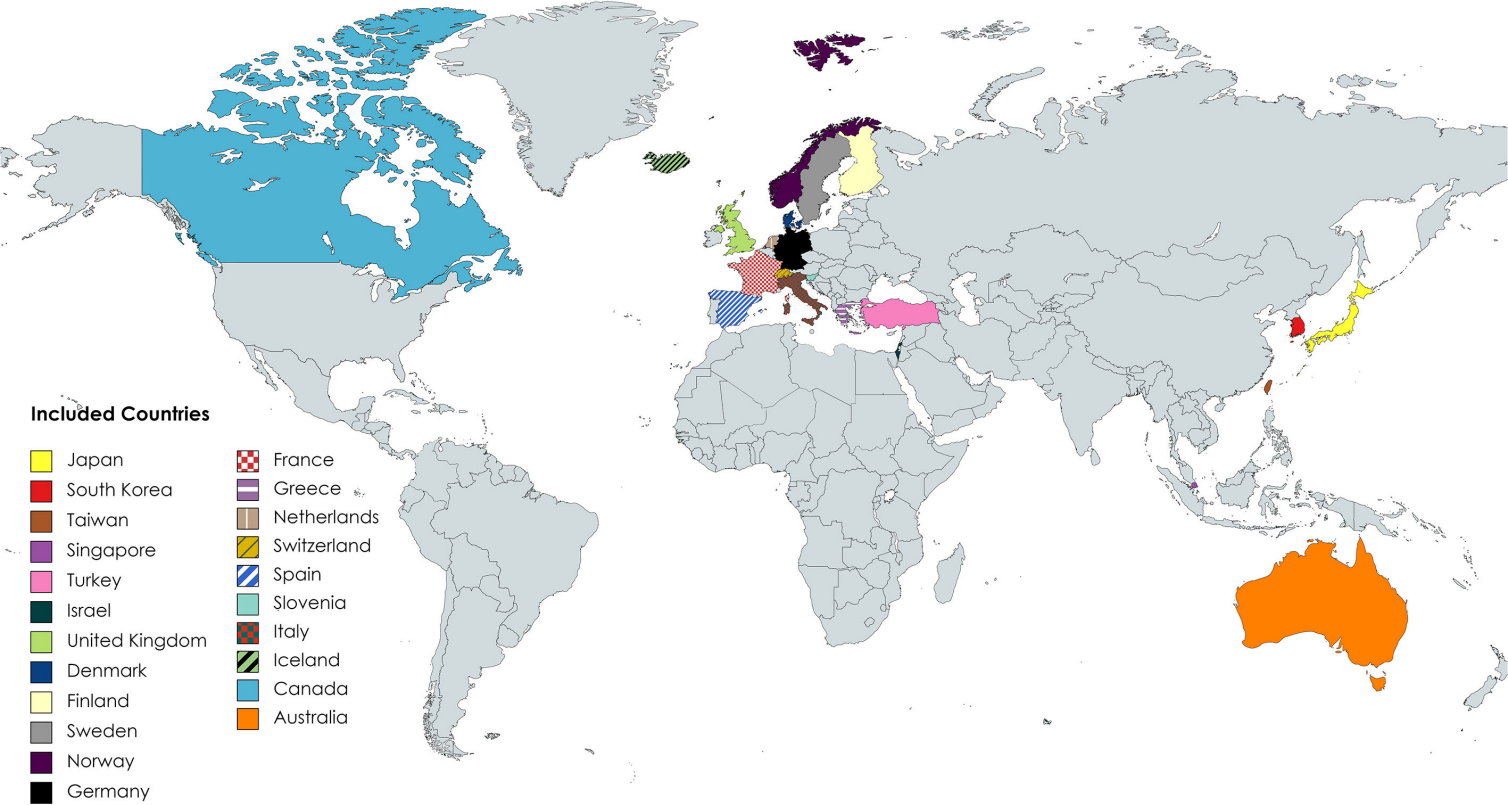
Countries of the included studies.

More than 2,434,975 cases of breast cancer [two studies did not mention the number of primary breast cancer patients (70, 73)] were included in the study, of which 91,678 patients developed second primary cancers. The summary of the characteristics of the included studies is shown in **Supplementary Table  1**. The design of all studies, except the study of An et al. (74), was a retrospective cohort. The study of An et al. was considered as a retrospective case–controlled study design. In terms of data source, three studies used hospital-based data (61, 63, 74), and the others used population-based data. A total of 16 studies mentioned that the IARC definition was used for defining the second primary cancers (10, 12, 14–17, 63, 66–69, 71–73, 77, 79), but the remaining studies did not mention this. In general, the studies included in this systematic review cover data from 22 countries: Japan, South Korea, Taiwan, Singapore, Turkey, Israel, United Kingdom, Finland, Denmark, Sweden, Norway, Germany, France, Greece, Netherlands, Switzerland, Spain, Slovenia, Italy, Iceland, Canada, and Australia (**Figure 2**). Furthermore, most of the studies were conducted on women, except the studies of Hung et al. (75) and AIRTUM Working Group (14), which were conducted on both sexes, and the study of Hemminki et al. (66), which was conducted on men. The methodological quality of the included articles was evaluated as high, except for the articles that were prone to misclassification bias (61, 68) and/or selection bias (15, 17, 59, 61, 62, 70, 73, 74, 77).

### Results of the meta-analysis

**Figure 3:**
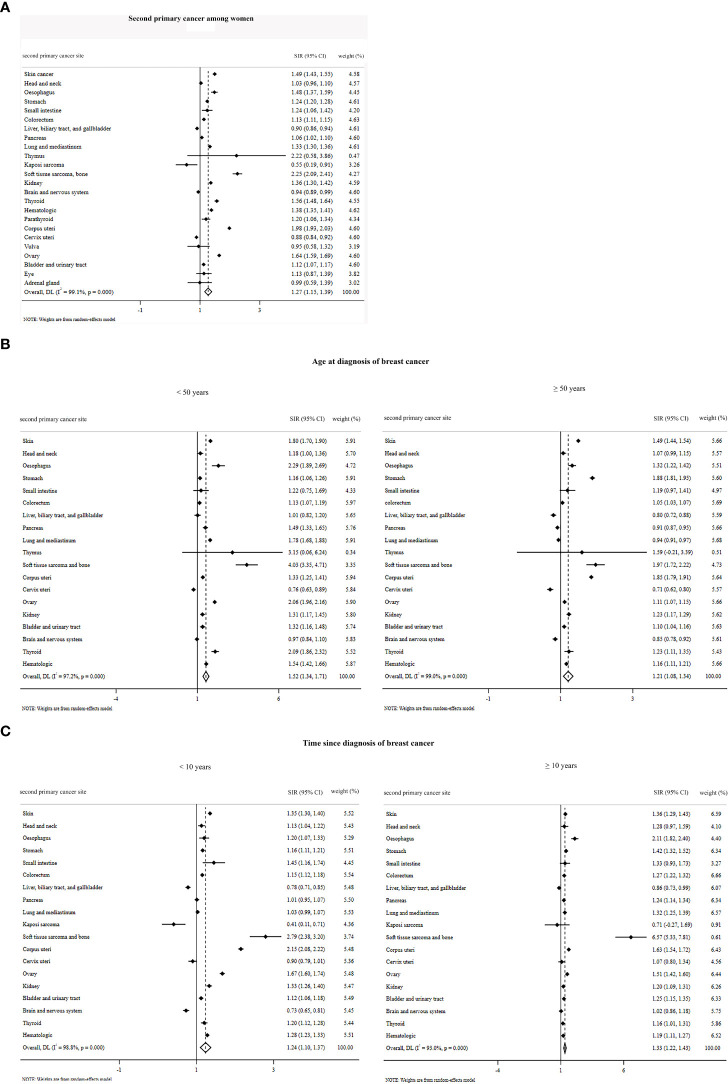
Overall standardized incidence ratios of second primary cancer among women **(A)** by age at diagnosis of breast cancer **(B)** and by time since the diagnosis of breast cancer **(C)**.

**Figure 4:**
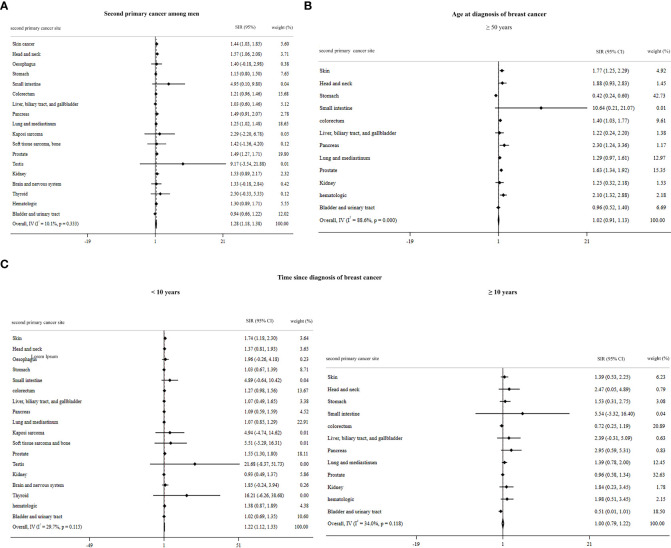
Overall standardized incidence ratios of second primary cancer among men **(A)** by age at diagnosis of breast cancer **(B)** and by time since the diagnosis of breast cancer **(C)**.

The summary of pooled estimates based on sex, second primary cancer site, age at diagnosis of breast cancer, and time since breast cancer diagnosis is provided in **Tables 1**–**4**. The summarized SIR estimate for female patients (**Figure 3A**) and male patients (**Figure 4A**) was 1.27 (95% CI: 1.15, 1.39) and 1.28 (95% CI: 1.18, 1.38), respectively.

**Table 1:** Standardized incidence ratios (SIRs) of second cancers by second cancer site in female and male patients.

Second primary cancer site	Female					Male				
	Observed	SIR	95% CI	Number of studies/Ref.	*I* ^2^ value	Observed	SIR	95% CI	Number of studies/Ref.	*I* ^2^ value
Skin cancer	2,399	1.49^a^	1.30–1.67	12/ (12, 14–17, 27, 61, 64, 67, 68, 71, 75)	85.8%	47	1.44^a^	1.03–1.86	3/ (14, 66, 75)	49%
Melanoma skin cancer	2,234	1.37^a^	1.26–1.47	9/ (14–17, 27, 61, 64, 68, 71)	54.6%	11	1.04	0.4–1.69	2/ (14, 66)	0%
Non-melanoma skin cancer	165	2.87	0.18–5.57	2/ (12, 67)	97.2%	36	1.65^a^	1.16–2.29	1/ (66)	0%
Head and neck	929	1.03	0.85–1.22	10/ (14, 17, 59, 61, 64, 67, 68, 71, 75, 76)	76.1%	37	1.57	0.57–2.56	3/ (14, 66, 75)	67.2%
Oral cavity	520	1.21^a^	1.02–1.40	5/ (14, 17, 59, 61, 71)	44.9%	4	2.1	–	1/ (14)	–
Pharynx	123	1.06	0.88–1.25	2/ (14, 71)	0%	3	1.78	–	1/ (14)	–
Larynx	105	1.01	0.82–1.21	3/ (14, 61, 71)	0%	12	1.10	0.48–1.72	2/ (14, 66)	0%
Esophagus	678	1.48^a^	1.28–1.69	11/ (14, 17, 27, 59, 61, 64, 67, 68, 71, 75, 76)	51.6%	3	1.4	–	1/ (14)	–
Stomach	3,485	1.24^a^	1.13–1.35	13/ (12, 14, 17, 27, 59–61, 63–65, 67, 71, 75)	70.6%	41	1.15	0.80–1.51	3/ (14, 66, 75)	0%
Small intestine	189	1.24^a^	1.06–1.42	3/ (27, 67, 71)	0%	4	4.95^a^	1.35–12.7	1/ (66)	–
Colorectum	11,317	1.13^a^	1.07–1.19	16/ (12, 14–17, 27, 59–61, 63, 64, 67, 68, 71, 75, 76)	77.6%	93	1.21	0.96–1.46	3/ (14, 66, 75)	49.6%
Liver, biliary tract, and gallbladder	2,005	0.90	0.78–1.02	10/ (14, 27, 59, 60, 63, 64, 67, 68, 71, 75)	82.5%	22	1.03	0.60–1.47	3/ (14, 66, 75)	0%
Pancreas	2346	1.06^a^	1.02–1.11	13/ (14, 15, 17, 27, 59–61, 63, 64, 67, 68, 71, 75)	19.7%	25	1.49	0.88–2.10	3/ (14, 66, 75)	0%
Lung and mediastinum	5,840	1.33^a^	1.21–1.45	15/ (14–17, 27, 59, 61, 63, 64, 67, 68, 71, 73, 75, 76)	87.2%	118	1.25^a^	1.02–1.48	3/ (14, 66, 75)	0%
Thymus	7	2.22	0.89–4.56	1/ (67)	–	–	–	–	–	–
Kaposi sarcoma	9	0.55	–	1/ (14)	–	1	2.29	–	1/ (14)	–
Soft tissue sarcoma, bone	753	2.25^a^	1.79–2.70	10/ (14, 27, 61, 64, 67, 68, 71, 72, 75, 76)	84.5%	1	1.42	–	1/ (14)	–
Soft tissue sarcoma	612	2.17^a^	1.69–2.65	9/ (14, 27, 61, 64, 67, 68, 71, 72, 76)	82.3%	1	1.42	–	1/ (14)	–
Bone	103	1.68	0.98–2.39	4/ (14, 27, 67, 71)	75.5%	–	–	–	–	–
Prostate	–	–	–	–	–	178	1.49^a^	1.27–1.71	3/ (14, 66, 75)	12.6%
Testis	–	–	–	–	–	2	9.17^a^	–	1/ (14)	–
Kidney	2,302	1.36^a^	1.2–1.51	13/ (12, 14, 15, 17, 27, 60, 61, 64, 67, 68, 71, 75, 79)	78.1%	22	1.53	0.03–3.04	2/ (14, 66)	79.7%
Brain and nervous system	1,434	0.94	0.79–1.08	8/ (14, 16, 17, 27, 61, 67, 68, 71)	81.2%	3	1.33	–	1/ (14)	–
Thyroid	1,602	1.56^a^	1.40–1.71	18/ (12, 14–17, 27, 59, 61, 63–65, 67, 68, 71, 73–76)	62.8%	3	2.50	-0.63–5.63	2/ (14, 75)	0%
Hematologic	7,692	1.38^a^	1.19–1.52	18/ (12, 14–17, 27, 28, 59–61, 63, 64, 67, 68, 71, 75, 76, 78)	96.2%	38	1.30	0.11–2.49	3/ (14, 66, 75)	84.5%
Leukemia	1,901	1.43^a^	1.29–1.58	11/ (14, 16, 17, 27, 28, 59, 61, 64, 68, 71, 76)	65.4%	19	1.26	-0.49–3.01	2/ (14, 66)	88.3%
Lymphoma	3,301	1.10^a^	1.01–1.19	12/ (14–17, 27, 61, 63, 64, 68, 71, 76, 78)	70.2%	15	0.97	0.46–1.49	2/ (14, 66)	28.6%
Hodgkin’s lymphoma	75	1.08	0.84–1.33	2/ (14, 27)	0%	–	–	–	–	–
Non-Hodgkin’s lymphoma	1,330	1.15^a^	1.03–1.27	7/ (14, 16, 17, 27, 63, 64, 68)	58.7%	4	0.70	–	1/ (14)	–
Multiple myeloma	1,270	1.06	0.86–1.26	6/ (14, 27, 64, 68, 71, 78)	88.6%	1	0.37	–	1/ (14)	–
Parathyroid	272	1.20^a^	1.08–1.38	1/ (70)	–	–	–	–	–	–
Corpus uteri	7,009	1.98^a^	1.76–2.20	19/ (12, 14–17, 27, 59–61, 63, 64, 67–69, 71, 73, 75, 77, 79)	95.1%	–	–	–	–	–
Cervix uteri	1,566	0.88	0.83–0.92	13/ (14, 16, 27, 59, 60, 63, 64, 67–69, 71, 75, 79)	46.4%	–	–	–	–	–
Vulva	25	0.95	0.62–1.42	1/ (68)	–	–	–	–	–	–
Ovary	4,852	1.64^a^	1.49–1.78	21/ (12, 14–17, 27, 58–61, 63, 64, 67–69, 71, 73, 75–77, 79)	85.1%	–	–	–	–	–
Bladder and urinary tract	2,002	1.12^a^	1.07–1.17	13/ (12, 14, 17, 59–61, 63, 64, 67, 68, 71, 75, 79)	0%	43	0.94	0.66–1.22	3/ (14, 66, 75)	0%
Eye	75	1.13	–	1/ (71)	–	–	–	–	–	–
Adrenal gland	23	0.99	–	1/ (71)	–	–	–	–	–	–
All sites combined	58,811	1.27^a^	1.15–1.39	All above estimates	99.1%	718	1.28^a^	1.18–1.38	All above estimates	10.1%

a The 95% CI does not include 1.00.

**Table 2:** Standardized incidence ratios (SIRs) of second cancers by second cancer site, age at diagnosis, and time since breast cancer diagnosis in male patients.

Second primary cancer site	Age at diagnosis			Time since breast cancer diagnosis							
	≥50 years			<10 years				≥10 years			
	Observed	SIR (95% CI)	Study ref.	Observed	SIR (95% CI)	Number of Studies/Ref.	*I* ^2^ value	Observed	SIR (95% CI)	Study ref.	*I* ^2^ value
Skin	45	1.77^a^ (1.28–244)	(66)	37	1.74^a^ (1.18–2.30)	(14, 66)	0%	10	1.39 (0.51–2.26)	(14, 66)	0%
Skin melanoma	9	1.71 (0.79–3.67)		8	1.54 (0.47–2.61)	2/ (14, 66)	0%	3	1.38 (-0.27–3.03)	(14, 66)	0%
Skin non- melanoma	36	1.78^a^ (1.25–2.54)		29	1.8^a^ (1.22–2.66)	1/ (66)	–	7	1.38 (0.56–2.85)	(66)	–
Head and neck	15	1.88^a^ (1.04–3.43)		23	1.37 (0.81–1.93)	2/ (14, 66)	0%	4	2.47 (0.04–4.91)	(14, 66)	0%
Oral cavity	–	–		3	1.5 (-0.4–3.39)	1/ (14)	–	1	5.02 (-5.02–15.34)	(14)	–
Pharynx	–	–		3	1.67 (-0.44–3.79)	1/ (14)	–	–	–	–	–
Larynx	7	3.04^a^ (1.3–7.14)		4	1.19 (-0.01–2.38)	1/ (14)	–	1	2.2 (-2.16–6.56)	(14)	–
Esophagus	–	–		3	1.96 (-0.27–4.19)	1/ (14)	–	–	–	–	–
Stomach	22	0.42 (0.14–0.71)		31	1.03 (0.67–1.39)	2/ (14, 66)	0%	6	1.53 (0.56–3.34)	(66)	–
Small intestine	4	10.64^a^ (3.24–34.95)		3	4.89^a^ (1.15–20.7)	1/ (66)	–	1	5.54 (0.14–30.9)	(66)	–
Colorectum	55	1.40^a^ (1.06–1.86)		74	1.27 (0.80–1.74)	2/ (14, 66)	61%	9	0.72 (0.25–1.19)	(14, 66)	0%
Liver, biliary tract, and gallbladder	6	1.22 (0.46–3.26)		13	1.07 (0.46–1.67)	2/ (14, 66)	3.4%	3	2.39 (-0.42–5.20)	(14)	–
Pancreas	18	2.30^a^ (1.36–3.87)		18	1.09 (0.02–2.17)	2/ (14, 66)	74.5%	6	2.95^a^ (1.08–6.43)	(66)	–
Lung and mediastinum	63	1.29 (0.99–1.68)		88	1.07 (0.84–1.29)	2/ (14, 66)	14.3%	20	1.39 (0.78–2.00)	(14, 66)	0%
Kaposi sarcoma	–	–		1	4.94	1/ (14)	–	–	–	–	–
Soft tissue sarcoma and bone	–	–		1	5.51	1/ (14)	–	–	–	–	–
Soft tissue sarcoma	–	–		1	5.51	1/ (14)	–	–	–	–	–
Bone	–	–		–	–	–	–	–	–	–	–
Prostate	119	1.63^a^ (1.35–1.97)		146	1.55^a^ (1.18–1.92)	2/ (14, 66)	52.9%	25	0.96 (0.34–1.57)	(14, 66)	52.4%
Testis	–	–		2	21.68 (-15.79–59.15)	1/ (14)	–	–	–	–	–
Kidney	7	1.25 (0.51–3.04)		17	0.93 (-0.56–2.42)	2/ (14, 66)	89.1%	5	1.84 (0.17–3.50)	(14, 66)	0%
Brain and nervous system	–	–		3	1.85 (-0.25–3.96)	1/ (14)	–	–	–	–	–
Thyroid	–	–		2	16.21^a^	1/ (14)	–	–	–	–	–
Hematologic	28	2.10^a^ (1.37–3.21)		28	1.38 (0.47–2.29)	2/ (14, 66)	66.1%	7	1.98 (0.84–4.66)	(66)	–
Leukemia	17	2.63^a^ (1.54–4.52)		16	2.21^a^ (1.11–3.30)	2/ (14, 66)	0%	3	1.78 (0.37–5.20)	(66)	–
Lymphoma	11	1.43 (0.71–2.88)		11	1.00 (0.40–1.60)	2/ (14, 66)	Model: fixed (I^2 =^ 0%)	4	2.14 (0.58–5.48)	(66)	–
Hodgkin’s lymphoma	–	–		–	–	–	–	–	–	–	–
Non- Hodgkin’s lymphoma	–	–		4	0.88 (-0.1–1.86)	1/ (14)	–	–	–	–	–
Multiple myeloma	–	–		1	0.80	1/ (14)	–	–	–	–	–
Bladder and urinary tract	18	0.96 (0.57–1.61)		37	1.02 (0.69–1.34)	2/ (14, 66)	Model: fixed (I^2 =^ 0%)	4	0.51 (0.00–1.03)	(14, 66)	0%
All sites combined	400	1.02 (0.91–1.13)	All above estimates using fixed effect model (I^2^ value= 88.6%)	527	1.22^a^ (1.12–1.33)	All above estimates	29.7%	100	1.00 (0.79–1.22)	All above estimates	34.0%

a The 95% CI does not include 1.00.

**Table 3:** Standardized incidence ratios (SIRs) of second cancers by site and age at diagnosis in female patients.

Second primary cancer site	Age at diagnosis							
	<50 years				≥50 years			
	Observed	SIR (95% CI)	No. of studies/Ref.	*I* ^2^ value	Observed	SIR (95% CI)	No. of studies/Ref.	*I* ^2^ value
Skin	1,196.5	1.80^a^ (1.29, 2.31)	6/ (10, 12, 27, 61, 62, 67)	85%	3,986.5	1.49^a^ (1.20, 1.78)	6/ (10, 12, 27, 61, 62, 67)	90.1%
•Skin melanoma	482	1.33^a^ (1.21, 1.45)	4/ (10, 27, 61, 62)	I^2 =^ 0%	1,181	1.28^a^ (1.20, 1.45)	4/ (10, 27, 61, 62)	I^2 =^ 0%
•Skin non-melanoma	774.5	4.12^a^ (1.52, 6.72)	3/ (10, 12, 67)	90.6%	2,805.5	1.97^a^ (1.14, 2.81)	3/ (10, 12, 67)	94.4%
Head and neck	168	1.18^a^ (1.00, 1.37)	5/ (10, 59, 61, 62, 67)	0%	628	1.07 (0.74, 1.39)	5/ (10, 59, 61, 62, 67)	74.8%
Oral cavity	12.5	1.15 (0.45, 1.85)	3/ (59, 61, 62)	11.9%	33.5	0.76 (0.49, 1.02)	3/ (59, 61, 62)	0%
Pharynx	–	–	–	–	–	–	–	–
Larynx	26	1.18 (0.72, 1.64)	2/ (10, 62)	0%	98	1.11 (0.54, 1.68)	2/ (10, 62)	81.4%
Esophagus	127.5	2.29^a^ (1.89, 2.69)	6/ (10, 27, 59, 61, 62, 67)	0%	622.5	1.32^a^ (1.21, 1.42)	6/ (10, 27, 59, 61, 62, 67)	38.4%
Stomach	537	1.16^a^ (1.00, 1.32)	8/ (10, 12, 27, 59, 61–63, 67)	15.4%	2,570	1.88^a^ (1.72, 2.04)	8/ (10, 12, 27, 59, 61–63, 67)	81.1%
Small intestine	25.5	1.22 (0.64, 1.80)	2/ (10, 67)	0%	117.5	1.19 (0.73, 1.66)	2/ (10, 67)	50.4%
Colorectum	1,348	1.13 (0.94, 1.32)	9/ (10, 12, 27, 59–63, 67)	68.3%	7,288	1.05 (0.92, 1.18)	9/ (10, 12, 27, 59–63, 67)	91.3%
Liver, biliary tract, and gallbladder	107	1.01^a^ (0.81, 1.20)	5/ (10, 59, 62, 63, 67)	0%	419	0.80 (0.72, 0.88)	(10, 59, 62, 63, 67)	46.3%
Pancreas	343	1.49^a^ (1.33, 1.65)	6/ (10, 27, 59, 62, 63, 67)	0%	1,572	0.91 (0.73, 1.09)	6/ (10, 27, 59, 62, 63, 67)	84.2%
Lung and mediastinum	1,221.5	1.78^a^ (1.44, 2.13)	7/ (10, 27, 59, 61–63, 67)	78.2%	3,212.5	0.94 (0.68, 1.19)	7/ (10, 27, 59, 61–63, 67)	96.6%
Kaposi sarcoma	–	–	–	–	–	–	–	–
Thymus	4	3.15 (0.85, 8.06)	1/ (67)	–	3	1.59 (0.32, 4.64)	1/ (67)	–
Soft tissue sarcoma and bone	136	4.03^a^ (2.43, 5.63)	5/ (10, 27, 61, 62, 67)	74.8%	231	1.97 (0.97, 297)	5/ (10, 27, 61, 62, 67)	92.0%
•Soft tissue sarcoma	99	4.09^a^ (1.92, 6.25)	5/ (10, 27, 61, 62, 67)	82.0%	191	2.34^a^ (1.14, 3.55)	5/ (10, 27, 61, 62, 67)	91.6%
•Bone	37	3.02^a^ (2.02, 4.03)	3/ (10, 62, 67)	12.2%	40	1.04 (0.20, 1.88)	3/ (10, 62, 67)	73.7%
Corpus uteri	1,041	1.33^a^ (1.10, 1.56)	11/ (10, 12, 27, 59–63, 67, 77, 79)	73.2%	4,091	1.85^a^ (1.62, 2.09)	11/ (10, 12, 27, 59–63, 67, 77, 79)	90.2%
Cervix uteri	133	0.76 (0.63, 0.90)	6/ (59, 60, 62, 63, 67, 79)	25.8%	258	0.71 (0.51, 0.86)	6/ (59, 60, 62, 63, 67, 79)	50.5%
Ovary	1,507.5	2.06^a^ (1.76, 2.36)	12/ (10, 12, 27, 58–63, 67, 77, 79)	72.8%	2,547.5	1.11 (0.95, 1.27)	12/ (10, 12, 27, 58–63, 67, 77, 79)	78.52%
Kidney	320	1.31^a^ (1.17, 1.46)	7/ (10, 12, 27, 61, 62, 67, 79)	19.0%	1,387	1.23^a^ (1.17, 1.30)	7/ (10, 12, 27, 61, 62, 67, 79)	45.5%
Bladder and urinary tract	246	1.32^a^ (1.16, 1.49)	8/ (10, 12, 59, 61–63, 67, 79)	0%	1,320	1.10^a^ (1.04, 1.16)	8/ (10, 12, 59, 61–63, 67, 79)	32.1%
Brain and nervous system	205	0.97 (0.84, 1.11)	5/ (10, 27, 61, 62, 67)	39.4%	546	0.85 (0.59, 1.12)	4/ (10, 27, 62, 67)	88.6%
Thyroid	324	2.09^a^ (1.86, 2.31)	9/ (10, 12, 27, 59, 61–63, 65, 67)	0%	376	1.23 (0.95, 1.51)	8/ (10, 27, 59, 61–63, 65, 67)	62.2%
Hematologic	660	1.54^a^ (1.42, 1.66)	9/ (10, 12, 27, 59–63, 67)	0%	2,533	1.16 (0.96, 1.35)	9/ (10, 12, 27, 59–63, 67)	62.2%
•Leukemia	319.5	1.91^a^ (1.70, 2.12)	5/ (10, 27, 59, 61, 62)	0%	1,263.5	1.34^a^ (1.15, 1.52)	5/ (10, 27, 59, 61, 62)	65.7%
•Lymphoma	311.5	1.24^a^ (1.10,1.38)	5/ (10, 27, 61–63)	44.00%	1,177.5	1.03 (0.82, 1.24)	5/ (10, 27, 61–63)	83.6%
•Hodgkin’s lymphoma	–	–	–	–	–	–	–	–
•Non-Hodgkin’s lymphoma	309.5	1.25 (0.96, 1.54)	4/ (10, 27, 62, 63)	58.0%	1,169.5	1.01 (0.80, 1.22)	4/ (10, 27, 62, 63)	87.0%
•Multiple myeloma	9	1.01 (0.52, 1.94)	1/ (62)	–	55	0.63 (0.48, 0.82)	1/ (62)	–
All sites combined	9,650.5	1.52^a^ (1.34, 1.71)	All above estimates	97.2%	33,736.5	1.21^a^ (1.08, 1.34)	All above estimates	99.0%

a The 95% CI does not include 1.00.

**Table 4:** Standardized incidence ratios (SIRs) of second cancers by second cancer size and time since the diagnosis of breast cancer in female patients.

Second primary cancer site	Time since the diagnosis of breast cancer							
	<10 years				≥10 years			
	Observed	SIR (95% CI)	No. of studies/Ref.	*I* ^2^ value	Observed	SIR (95% CI)	No. of studies/Ref.	*I* ^2^ value
Skin	3,236	1.35 (0.99, 1.71)	4/ (10, 14, 61, 67)	90.4%	1,625	1.36 (0.87, 1.85)	4/ (10, 14, 61, 67)	77.5%
•Skin melanoma	1,083	1.19^a^ (1.05, 1.33)	3/ (10, 14, 61)	56.1%	407	1.37^a^ (1.24, 1.50)	3/ (10, 14, 61)	29.7%
•Skin non-melanoma	2,152	3.72 (-1.24, 8.69)	2/ (10, 67)	87.0%	1,218	1.28 (0.16, 2.39)	2/ (10, 67)	85.3%
Head and neck	626	1.13 (0.94, 1.32)	4/ (10, 14, 59, 61)	50.4%	64	1.28 (0.96, 1.60)	3/ (10, 14, 59)	0%
Oral cavity	93	1.07 (0.85, 1.29)	3/ (14, 59, 61)	29.2%	21	1.21 (0.68, 1.75)	2/ (14, 59)	20.6%
Pharynx	36	0.98 (0.65, 1.31)	1/ (14)	–	9	1.48 (0.51, 2.45)	1/ (14)	–
Larynx	113	1.18 (0.65, 1.71)	3/ (10, 14, 61)	71.1%	33	1.15 (0.76, 1.55)	2/ (10, 14)	0%
Esophagus	331	1.20^a^ (1.07, 1.34)	4/ (10, 14, 61, 67)	0%	210	2.11^a^ (1.83, 2.40)	3/ (10, 14, 67)	0%
Stomach	2,044	1.16 (0.95, 1.37)	5/ (10, 14, 59, 61, 63)	86.6%	744	1.42^a^ (1.32, 1.52)	5/ (10, 14, 59, 61, 63)	32.7%
Small intestine	97	1.45^a^ (1.18, 1.78)	1/ (10)	–	42	1.33 (0.96, 1.8)	1/ (10)	–
Colorectum	5,470	1.15^a^ (1.05, 1.24)	5/ (10, 14, 59, 61, 63)	69.6%	2,284	1.27^a^ (1.22, 1.32)	5/ (10, 14, 59, 61, 63)	37.4%
Liver, biliary tract, and gallbladder	516	0.78 (0.71, 0.84)	3/ (10, 14, 63)	0%	163	086 (0.72, 0.99)	3/ (10, 14, 63)	0%
Pancreas	1,169	1.01 (0.95, 1.07)	3/ (10, 14, 61)	0%	574	1.24^a^ (1.03, 1.44)	2/ (10, 14)	63.5%
Lung and mediastinum	2,545	1.03 (0.87, 1.19)	6/ (10, 14, 59, 61, 63, 67)	78.2%	1,399	1.32 (0.91, 1.74)	6/ (10, 14, 59, 61, 63, 67)	86.8%
Kaposi sarcoma	7	0.41 (0.07, 0.75)	1/ (14)	–	2	0.71 (-0.28, 1.7)	1/ (14)	–
Thymus -								
Soft tissue sarcoma and bone	182	2.79 (0.84, 4.73)	4/ (10, 14, 61, 67)	92.2%	108	4.94 (0.41, 9.46)	3/ (10, 14, 61)	Model: random (I^2^=94.2%)
•Soft tissue sarcoma	130	4.06 (0.77, 7.34)	3/ (10, 14, 61)	93.9%	78	6.57 (-0.59, 13.72)	3/ (10, 14, 61)	Model: random (I^2^=94.3%)
•Bone	52	1.20 (0.30, 2.10)	3/ (10, 14, 67)	74.2%	30	2.43^a^ (1.55, 3.31)	2/ (10, 14)	Model: fixed (I^2^=0%)
Corpus uteri	3,770	2.15^a^ (1.81, 2.50)	7/ (10, 14, 61, 63, 67, 77, 79)	95.0%	1,246	1.63^a^ (1.32, 1.95)	7/ (10, 14, 61, 63, 67, 77, 79)	84.5%
Cervix uteri	262	0.90 (0.79, 1.01)	3/ (14, 63, 79)	27.5%	60	1.07 (0.79, 1.34)	2/ (14, 79)	0%
Ovary	2,479	1.67^a^ (1.42, 1.92)	7/ (10, 14, 61, 63, 67, 77, 79)	88.3%	1,111	1.51^a^ (1.21, 1.80)	7/ (10, 14, 61, 63, 67, 77, 79)	77.0%
Kidney	1,300	1.33^a^ (1.25, 1.40)	5/ (10, 14, 61, 67, 79)	41.3%	442	1.20^a^ (1.09, 1.31)	5/ (10, 14, 61, 67, 79)	17.6%
Bladder and urinary tract	1,319	1.12^a^ (1.06, 1.18)	5/ (10, 14, 61, 63, 79)	0%	566	1.25^a^ (1.14, 1.35)	4/ (10, 14, 61, 79)	0%
Brain and nervous system	315	0.73 (0.65, 0.81)	2/ (10, 14)	0%	149	1.02 (0.86, 1.19)	3/ (10, 14, 61)	0%
Thyroid	776	1.20^a^ (1.69, 2.72)	7/ (10, 14, 59, 61, 63, 65, 67)	84.5%	220	1.16 (0.61, 1.72)	5/ (10, 14, 59, 65, 67)	85.5%
Hematologic	2,339	1.28 (0.96, 1.60)	5/ (10, 14, 61, 63, 67)	92.7%	920	1.19 (0.86, 1.51)	5/ (10, 14, 61, 63, 67)	78.3% )
•Leukemia	1,087	1.44^a^ (1.11, 1.78)	3/ (10, 14, 61)	87.8%	376	1.30 (0.93, 1.67)	3/ (10, 14, 61)	68.7%
•Lymphoma	1,073	0.99 (0.86, 1.11)	4/ (10, 14, 61, 63)	52.7%	500	1.33^a^ (1.21, 1.44)	4/ (10, 14, 61, 63)	18.7%
•Hodgkin’s lymphoma	31	0.86 (0.52, 1.2)	1/ (14)	–	6	1.35 (0.27, 2.43)	1/ (14)	–
•Non-Hodgkin’s lymphoma	1,035	0.98 (0.86, 1.10)	3/ (10, 14, 63)	61.5%	491	1.33^a^ (1.21, 1.45)	3/ (10, 14, 63)	49.7%
•Multiple myeloma	166	0.9 (0.76, 1.04)	1/ (14)	–	43	1.17 (0.82, 1.52)	1/ (14)	–
All sites combined	28,783	1.24^a^ (1.10, 1.37)	All above estimates	98.8%	11,929	1.33^a^ (1.22, 1.43)	All above estimates	93.0%

a The 95% CI does not include 1.00.

Where the age at diagnosis is concerned, the pooled estimate for men older than 50 years using the fixed-effect model (although the *I* ^2^ value is higher than 50%, because only the estimates of one article (66) were pooled, the fixed model was used) was 1.02 (95% CI: 0.91, 1.13). Moreover, the SIR by time since diagnosis of breast cancer for male patients <10 years and ≥10 years was 1.22 (95% CI: 1.12, 1.33) and 1.00 (95% CI: 0.79, 1.22), respectively (**Figures 4B, C**). The overall SIR by age at diagnosis of breast cancer for female patients <50 years and ≥50 years was 1.52 (95% CI: 1.34, 1.71) and 1.21 (95% CI: 1.08, 1.34), respectively (**Figure 3B**). The overall SIR by time since diagnosis of breast cancer for female patients <10 years and ≥10 years was 1.24 (95% CI: 1.10, 1.37) and 1.33 (95% CI: 1.22, 1.43), respectively (**Figure 3C**).

Publication bias was seen in the following second primary cancers: among female patients based on second cancer sites—melanoma skin cancer [trim-and-fill method: 1.35 (95% CI: 1.23, 1.47)], soft tissue sarcoma and bone [trim-and-fill method: 1.87 (95% CI: 1.35, 2.39)], soft tissue sarcoma [trim-and-fill method: 2.16 (95% CI: 12.65, 2.66)], non-Hodgkin’s lymphoma [trim-and-fill method: 1.11 (95% CI: 0.99, 1.23)], corpus uteri [trim-and-fill method: 1.74 (95% CI: 1.54, 1.95)], and ovary [trim-and-fill method: 1.49 (95% CI: 1.33, 1.64)]; among female patients younger than 50 years—esophagus [trim-and-fill method: 2.27 (95% CI: 1.87, 2.66)]; and among female patients with second cancer diagnosis less than 10 years from breast cancer diagnosis—thyroid [trim-and-fill method: 2.20 (95% CI: 1.69, 2.72)] and kidney [trim-and-fill method: 1.32 (95% CI: 1.25, 1.40)].

The meta-regression analysis revealed that study quality only influences the risk estimate in the bladder and urinary tract cancer among female patients ≥50 years (regression coefficient = 0.2178597, *P* = 0.037).

In the sensitivity analysis, only the second primary cancer sites described below depended on a certain study. Based on the second cancer site among female patients, the SIRs of the liver and gallbladder and biliary tract were dependent on the study of Hung et al. (75). After omitting this study, the SIR and its association 95% CI were as follows: 0.85 (95% CI: 0.74, 0.96). Based on the second primary cancer site among female patients ≥50 years, the SIR for head and neck, soft tissue sarcoma and bone, thyroid, and hematologic cancers was dependent on the study of Evans et al. (62). After omitting this study, the SIR and its association 95% CI were as follows: head and neck—1.23 (95% CI: 1.13, 1.33), soft tissue sarcoma and bone—2.45 (95% CI: 1.04, 3.88), thyroid—1.30 (95% CI: 1.01, 1.59), and hematologic—1.23 (95% CI: 1.12, 1.34). Based on the second primary cancer site among female patients with a second cancer diagnosis less than 10 years after breast cancer diagnosis, the SIR of stomach cancer depended on the study of Tanaka et al. (63). After omitting this study, the SIR and its association 95% CI were 1.22 (95% CI: 1.01, 1.44). Furthermore, based on the second primary cancer site among female patients with a second primary cancer diagnosis ≥10 years after breast cancer diagnosis, the SIR of hematologic cancer depended on the study of Lee et al. (67). After omitting this study, the SIR and its association 95% CI were 1.31 (95% CI: 1.04, 1.59).

## Discussion

Our study demonstrated that breast cancer patients have a higher risk of having second primary cancer, and this risk varied across different cancer sites and were dependent on the time since and age at the diagnosis of primary breast cancer. The incidence of second cancer can be attributed not only to genetic and underlying causes but also to factors such as treatment complications, lifestyle (obesity, low exercise, and tobacco and alcohol use), immune function, hormonal imbalances, age, race, environmental factors, and shared etiologic factors (80, 81). Knowingly, all these factors—even the prognostic factors—among breast cancer patients contribute to susceptibility to a second primary cancer among these patients (82, 83). The risk of the second primary cancers differs for different cancer sites. Male breast cancer patients are at a higher risk of developing second primary colorectal, small intestine, pancreas, thyroid, kidney, and testis cancers (14, 66, 84). Female breast cancer patients are at a higher risk of developing second primary cancer of the endometrium, ovary, thyroid, lung, soft tissue sarcomas, leukemia, melanoma, stomach, and colorectum (9, 57, 61–64, 85–91). Many studies with the aim of trying to explain the susceptibility of breast cancer patients to these second primary cancers have been conducted. Some second primary cancers such as breast, lung, thyroid, esophagus, stomach, leukemia, and soft tissue sarcoma of the upper limbs and thorax are likely to develop due to being close to the radiotherapy site (10, 19, 92). The cytotoxic agents used in chemotherapy also increase the risk of leukemia after breast cancer. Genetic mutations such as mutations of BRCA, CDKN2A, PALB2, TP53, and CHEK2 genes are associated with second cancers of the ovary, breast, prostate, pancreas, melanoma, soft tissue sarcoma, osteosarcoma, leukemia, brain tumors, and others. A strong relationship between breast cancer and endometrial cancer has been proven, which can be attributed to breast cancer hormone therapy with tamoxifen and other factors, such as obesity and genetic mutations (10, 81, 93). The relationship between thyroid and breast cancer can be explained by predisposing factors such as genetics, obesity, hormonal factors, chemotherapy, low physical activity, alcohol, and radiotherapy (94). The etiological roles of obesity and alcohol and tobacco use in the development of second cancers are underestimated, while these factors play a greater role in the development of second primary cancers compared with genetics and treatment modalities. The most common cancers related to alcohol use include cancers of the oral cavity and pharynx, larynx, esophagus, liver, and breast. Moreover, obesity is a known risk factor for breast, colorectal, and kidney cancers (81, 93).

The present study is the first meta-analysis to investigate the risk of second cancers among women and men previously diagnosed with primary breast cancer by the site of second cancer, age at diagnosis of breast cancer, and time since diagnosis of breast cancer according to the IARC definition. Despite a large amount of research that has been done on the risk of developing second cancer among female breast cancer patients, few studies have been done on male breast cancer patients (45, 81, 95–98). According to the IARC definition and the inclusion criteria of this systematic review and meta-analysis, only three studies conducted among men (14, 66, 75) were eligible to be included in the study, which can reduce the reliability of the results among men. According to the results of the present study, breast cancer survivors among women and men have 27% and 28% extra risk of developing second primary cancers, respectively. This estimate is consistent with the reported risk in some previous studies among men (84) and women (10, 41, 59, 63). Based on our results and previous studies (7), premenopausal women (younger than 50 years) are at a higher risk of the occurrence of second cancers. Furthermore, the more time passes since breast cancer diagnosis, the more women are at risk of developing second primary cancer. However, based on the present results and the previous study (84), after 10 years from breast cancer diagnosis, men have a lower risk of developing second cancer compared with men diagnosed with breast cancer less than 10 years ago.

In the meta-analysis, in addition to high-quality studies, we also considered acceptable and low-quality studies. Meta-regression showed that the quality of the studies did not influence the estimated risk except in bladder and urinary tract cancer among female patients ≥50 years.

As a shortcoming of our study, researchers in this field are at risk of various types of bias due to differences among studies (such as populations, study designs, and settings) as well as unavoidable bias due to differences in the year the research was performed (due to advancement in breast cancer treatment and the possible change in the pattern of second cancers compared with previous decades). Therefore, we recommend that in the future, more research will be conducted that will also assess the mentioned factors.

## Conclusion

Results indicate a higher risk of developing second primary non-breast cancers among male and female breast cancer patients. Therefore, this population might benefit from prevention and screening programs. To prevent and control second primary cancers, the results of our systematic review can help researchers to design studies on screening modalities and prevention programs concerning this issue.

## Data availability statement

The original contributions presented in the study are included in the article/**Supplementary Material**. Further inquiries can be directed to the corresponding authors.

## Author contributions

PP, AM, MM, and AB researched, screened, and drafted the manuscript. AK and SH analyzed the data. AM and NA-Y supervised the content. All authors contributed to the article and approved the submitted version.
