# Supplementary material for: Risk of second primary cancer among breast cancer patients: A systematic review and meta-analysis
Source: Front Oncol. 2023 Jan 17;12:1094136. doi: 10.3389/fonc.2022.1094136 (PMC9887162; doi:10.3389/fonc.2022.1094136)
Supplement: Supplementary file 1 [file DataSheet_1.docx]

**Supplementary materials**

Supplementary Table 1 Characteristics of included studies

| Author publication year reference | Setting / source of data | Design  Study follow-up period | N first breast cancer/N second non breast cancer | Study population: definition and inclusion criteria | Definition of second primary cancers: inclusion criteria | Statistically significant SIRs for second cancer sites | Quality |
| --- | --- | --- | --- | --- | --- | --- | --- |
| Prior 1981 (1) | England/Birmingham and West Midlands Regional  Cancer Registry | Cohort  1950-1964  Follow-up until 1965 | 17,756/23 | Women older than 15 years with first primary breast cancer were included. | Only ovary cancer considered as second cancer.  SIR reported for: ovary  IARC coding: not said, but Birmingham and West Midlands Regional Cancer Registry follow IARC rules. | >45 y: ovary | High (++) |
| Murakami 1987 (2) | Osaka, Japan/ Osaka Cancer Registry | Cohort  1965-1982  Followed up until 1983 | 9,503/254 | Females with primary breast were included. | Second cancers defined as invasive tumors that occurred after 3 months of initial diagnosis.  SIRs reported for: all, all excluding breast, buccal cavity, esophagus, stomach, colon, rectum, liver, pancreas, lung breast, cervix uteri, corpus uteri, ovary, bladder, thyroid, and leukemia.  IARC coding: not said, but Osaka Cancer Registry follow IARC rules. | All: all, all excluding breast, buccal cavity, stomach, colon, thyroid, and breast.  >45 y: all, all excluding breast, buccal cavity, stomach, lung, breast.  45-54 y: all, all excluding breast, buccal cavity, stomach, breast.  ≥55y: all, thyroid, breast.  By time:  >1y: all, buccal cavity, breast.  1-4y: all, all excluding breast, stomach, breast, thyroid.  5-9y: all, breast.  ≥10y: all, all excluding breast, buccal cavity, lung, breast. | Acceptable (+)  Selection bias: Only women who were alive at the end of the study were included in the study. |
| Brenner 1993 (3) | Saarland, Germany/cancer registry of Saarland | Cohort 1968- 1987 | 9,678/206 | Woman diagnosed with breast cancer (ICD-9: 174) through physicians. | Second cancers diagnosed more than 1 year after first cancer. Following second tumours were excluded: unspecified location(ICD-9 195-199), liver (ICD-9 position 155), lung (ICD-9 162), bone (ICD-9 170), brain and nervous system (ICD-9 191-192), and skin tumours (ICD-9 172-173).  SIRs reported for: stomach, colon, rectum, gallbladder and bileducts, pancreas, cervix uteri, corpus uteri, ovary, urinary bladder, kidney, lymphoma, and leukemia.  IARC coding: not said, but cancer registry of Saarland follow IARC rules. | All: none  <50 y: ovary  ≥50 y: none | High (++) |
| Rubino 2000 (4) | France/the Institut Gustave Roussy | Cohort  1973-1992 | 4,416/193 | Woman survived more than one year following the first cancer were included. | Second cancers of bilateral breast cancer and non-melanoma skin cancer were not considered as second cancer. If a patient developed one of these two cancer were stayed in the study until the developing of a second malignancy, death or the end of study.  SIRs reported for: oral cavity, esophagus, stomach, colorectum, liver and gallbladder, pancreas, larynx, lung, corpus uteri, ovary, bladder, kidney, melanoma, nervous system, thyroid, other endocrine, bone, soft tissue, myeloma, lymphoma, leukemia, undefined sites, and all.  IARC coding: not said, but the Institut Gustave Roussy follows IARC rules. | All: ovary, bladder, melanoma, nervous system, soft tissue, leukemia, and all.  >50y: all, leukemia, soft tissue, melanoma, kidney, ovary, corpus uteri, lung, digestive tract, colorectal.  ≥50y: corpus uteri, kidney, soft tissue, leukemia, all.  By time:  >10y: corpus uteri, kidney, thyroid, soft tissue, leukemia, all.  ≥10 y: soft tissue, melanoma, ovary, corpus uteri, leukemia, all. | Low (-)  Misclassification bias:  it is not said whether first primary cancers were included. Selection bias: a high portion of patients were missed to follow-up. |
| Tanaka 2001 (5) | Osaka, Japan/Medical Center for Cancer and  Cardiovascular Diseases) | Cohort  1970–1994  Follow-up  until 1995 | 2,786/117 | Women aged between 20 and 75 years with a first primary invasive breast cancer (ICD-9, code 174) were included. | It is not said whether synchronous neoplasms were excluded. All second cancers (rather than non-melanoma skin cancer) were included.  SIRs reported for: all second cancers, Stomach, colon, rectum, liver, biliary tract, pancreas, lung, uterus, cervix, uterus corpus, ovary, bladder, thyroid gland, non-Hodgkin’s lymphoma.  IARC coding: yes | All: Ovary, thyroid and non-Hodgkin’s lymphoma.  >50 y: thyroid, non-Hodgkin’s lymphoma  ≥50 y: all second cancers | High (++) |
| Evan 2001 (6) | England/ The Thames Cancer Registry (TCR) | Cohort  1961-1995 | 145,677/4,470 | Resident women in the North or South Thames region diagnosed with first breast cancer during 1961-1995.  . | Following patients were excluded: non-melanoma skin cancers, non-malignant neoplasms, second neoplasm occurring less than after first cancer, same laterality and histology, diagnosis of two cancers at different sites on the same day.  SIRs reported for: tongue, mouth, esophagus, stomach, colon, rectum, liver, gallbladder, pancreas, larynx, lung, bone, connective tissues, skin melanoma, breast, cervix uteri, corpus uteri, ovary, bladder, kidney, brain and nervous system, thyroid, non-Hodgkin lymphoma, multiple myeloma, lymphoid and myeloid leukemia, all sites, and all sites excluding breast cancer.  IARC coding: not said, but Thames Cancer Registry follow IARC rules. | <50 y: esophagus, stomach, lung, bone, connective tissues, breast, cervix uteri, corpus uteri, ovary, myeloid leukemia, all sites, and all sites excluding breast cancer.  ≥50 y: Colon, liver, pancreas, lung, breast, cervix uteri, corpus uteri, brain and nervous system, non-Hodgkin lymphoma, multiple myeloma, myeloid leukemia, all sites, and all sites excluding breast cancer. | Acceptable (+)  Selection bias: women with unknown  data on date of diagnosis or on residence were excluded. |
| Levi 2003 (7) | Switzerland/Swiss Cancer Registries of Vaud and Neuchâtel | Cohort  1974-1998 | 9,724/443 | Women with invasive breast cancer were included. | Patients with less than 1 month of follow-up and synchronous Cancers were excluded.  SIRs reported for: mouth or pharynx, esophagus, stomach, colorectum, gallbladder, pancreas, lung, soft tissue, skin melanoma, cervix uteri, corpus uteri, ovary, other genital female organ, bladder, kidney, thyroid, non-Hodgkin’s lymphoma, multiple myeloma, leukemia.  IARC coding: not said, but Swiss Cancer Registries of Vaud and Neuchâtel follow IARC rules. | All: soft tissue, corpus uteri | High (++) |
| Sadetzki 2003 (8) | Israel/Israel  Cancer Registry | Cohort  1960-1998 | 49,207/59 | Breast cancer patients as coded in the ICD-9 (.0–174.9) were included. | Second primary cancer defined as cancer diagnosed at least one year after the first primary cancer.  SIR reported for: thyroid  IARC coding: not said, but Israel Cancer Registry follow IARC rules. | All: thyroid | High (++) |
| Hemminki 2005 (9) | Cancer registries of British Columbia, Manitoba and Saskatchewan (Canada), Singapore,  Slovenia, Norway, Denmark, Scotland, New South Wales (Australia),  Sweden, Finland, Iceland, and Zaragoza (Spain) | Cohort  1943-1998 | 3,409/426 | Males with first primary breast cancer were included. | Based on data of each centers, second tumors were included.  SIR reported for: all malignancies, oral cavity, stomach, small intestine, colorectal, liver & gallbladder& bile duct, pancreas, larynx, lung, melanoma of skin, other neoplasm of skin, prostate, bladder, kidney, lymphomas, multiple myelomas, and leukemia.  IARC coding: yes | All: all malignancies, small intestine, colorectal, pancreas, other neoplasm of skin, prostate, leukemia, myeloid leukemia  56> y:  All malignancies, stomach, pancreas, lung.  56-65y: larynx, leukemia, myeloid leukemia.  66-74y: all malignancies, small intestine, other neoplasm of skin, and prostate.  >75y: all malignancies, colorectal, other neoplasm of skin, lymphohaematopoietic, lymphoid leukemia.  By time of diagnosis:  <1y: all malignancies, other neoplasm of skin, prostate.  1-9y: all malignancies, rectum, liver, prostate, lymphohaematopoietic, leukemia, myeloid leukemia.  <10y: pancreas | High (++) |
| Mellemkjær 2006 (10) | 13 Cancer Registries of Europe (Denmark,  Finland, Iceland, Norway,  Slovenia, Sweden)  Canada, Australia,  Singapore | Cohort  1943-2000 | 525,527/31,399 | Women diagnosed with a first primary breast (ICD-9, code 174) cancer were included. | Second malignancies diagnosed more than 3 months after the first breast cancer diagnosis were included.  SIRs reported for: Oral cavity, pharynx, esophagus, stomach,  small intestine, colorectal, liver, pancreas, larynx, lung, bone, soft  tissue, sarcoma, melanoma, non-melanoma skin, corpus uteri, ovary, bladder, kidney, brain, nervous system, thyroid gland, non-Hodgkin lymphoma, leukemia, myeloid leukemia.  IARC rules: yes | All: oral cavity and pharynx, esophagus, stomach, small intestine, colorectal,  pancreas, larynx, lung, bone, soft tissue, melanoma, non-melanoma, corpus uteri, ovary, other female organs, bladder, kidney, thyroid, non-Hodgkin lymphoma, leukemia,  myeloid leukemia  ≤45 y: esophagus, stomach, colorectal, pancreas, lung, bone, soft tissue, melanoma, non-melanoma, corpus uteri, ovary, other female organs, bladder, kidney, thyroid, leukemia,  myeloid leukemia  46–55 y: oral cavity and pharynx, esophagus, stomach, colorectal, pancreas, lung, bone,  soft tissue, melanoma, non-melanoma, corpus uteri, ovary, other female organs, bladder,  kidney, thyroid, non-Hodgkin lymphoma, leukemia, myeloid leukemia  ≥56 y: oral cavity and pharynx, esophagus, stomach, small intestine, colorectal, larynx,  lung, soft tissue, melanoma, non-melanoma, corpus uteri, ovary, other female organs,  bladder, kidney, thyroid, non-Hodgkin lymphoma, leukemia, myeloid leukemia | High (++) |
| Prochazka 2006 (11) | Sweden/Swedish Cancer  Registry | Cohort  1958-2000 | 152,586/9,758 | Women diagnosed with an invasive first primary breast cancer and alive more than 1 month after the breast cancer diagnosis were included. | Second cancers (except non-melanoma) defined cancer diagnosed at least more than 1 month after the first breast cancer diagnosis.  SIRs reported for: Upper aero digestive tract, salivary glands, esophagus, stomach, small intestine, colorectum, liver, pancreas, lung, cervix, endometrium, ovary, kidney, urinary organs,  melanoma, nervous system, thyroid gland, endocrine glands, bone, connective tissue, non-Hodgkin’s lymphoma, Hodgkin’s disease, multiple myeloma, plasmocytoma, leukemia (Acute lymphatic leukemia, chronic lymphatic leukemia, acute myeloid leukemia, chronic myeloid leukemia).  IARC coding: not specified, but the Swedish cancer registry follow the IARC rules. | All: salivary glands, esophagus, stomach, small intestine, colorectum, pancreas, lung,  endometrium, ovary, Kidney, melanoma, nervous system, thyroid, endocrine glands, bone, connective tissue, non-Hodgkin’s lymphoma, leukemia.  <50y: esophagus, stomach, colorectum, pancreas, lung, endometrium, ovary, kidney,  melanoma, thyroid, endocrine glands, connective tissue, thorax and upper limbs, non-Hodgkin’s lymphoma, acute myeloid leukemia, chronic myeloid leukemia.  ≥50 y: esophagus, stomach, colorectum, lung, endometrium, kidney, melanoma, nervous system, thyroid, connective tissue, thorax and upper limbs, acute lymphatic leukemia, acute myeloid leukemia, chronic myeloid leukemia | High (++) |
| Lee 2008 (12) | Taiwan/ Taiwan National Cancer Registry (TNCR) | Cohort  1979-2003 | 53,783/1,085 | Women with primary breast cancer (ICD9, code 174) were included. | Second cancers (including all cancer except melanoma) diagnosed at least more than 1 month after the first breast cancer diagnosis.  Females who alive more than 1 month of breast cancer diagnosis were included.  SIRs reported for: Bone, corpus uteri, ovary, non-melanoma skin,  thyroid gland, head and neck, small intestine, colon and rectum,  liver, pancreas, lung, thymus, sarcoma, cervix uteri, urinary bladder, kidney and other urinary organs.  IARC coding: yes | All: bone, corpus uteri, ovary, non-melanoma, and thyroid  b50 y: bone, corpus uteri, ovary, oesophageal, kidney, lung, non-melanoma, leukemia or  lymphoma  ≥50 y: corpus uteri, ovary, cervix uteri, non-melanoma. | High (++) |
| Schaapveld 2008 (13) | Netherlands /Comprehensive Cancer Centers of the North  (Groningen), Amsterdam  and South (Eindhoven)  Netherlands, within the  Dutch Network of cancer registries). | Cohort  1989–2003  Follow-up until  2004 (for patients from the Comprehensive Cancer enter South) and 2005 (for the other registries) | 58,068/2,578 | Women diagnosed with a first primary breast cancer, without prior cancer (except non-melanoma skin cancer) were included. All unknown cancers were excluded. Synchronous and metachronous neoplasms were not excluded. | Non-melanoma skin cancer, meningioma, myelodysplastic syndrome, and polycythemia as second cancers were excluded. If a second cancer was diagnosed after a non-melanoma cancer, this cancer was included.  SIRs reported for: Head and neck, thyroid gland, esophagus, stomach, pancreas, gall bladder, extra hepatic bile ducts, colon, rectum, anus, lung, soft tissue, sarcomas, melanoma of skin, ovary, uterus, cervix, vulva, kidney, bladder, brain, leukemia, acute myeloid leukemia, non-Hodgkin’s lymphoma, multiple myeloma.  IARC coding: yes | All: esophagus, stomach, colon, rectum, lung, soft tissue, melanoma, uterus, ovary, bladder, kidney, non-Hodgkin’s lymphoma, acute myeloid leukemia.  >50 y: esophagus, rectum, ovary, uterus, soft tissue, melanoma, and acute myeloid leukemia.  50–69 y: melanoma, uterus, ovary, bladder, kidney, acute myeloid leukemia  ≥70 y: esophagus, soft tissue, melanoma, uterus, non-Hodgkin’s lymphoma. | Acceptable (+)  Misclassification bias:  Because synchronous and metachronous, was considered as the first primary breast cancer. |
| Verkooijen 2008 (14) | Switzerland/Geneva Cancer Registry | Cohort  1990-2004  Follow-up end: December 31, 2005 | 4,397/14 | Women with invasive breast cancer registered in Geneva Cancer Registry. | Second primary cancer defined as diagnosed cancer more than 6 months after first cancer.  SIRs reported for: chronic leukemia, acute leukemia, and all leukemia.  IARC coding: not said, but Geneva Cancer Registry follow IARC rules. | All: acute leukemia | High (++) |
| Gulhan 2009 (15) | Turkey/ Izmir cancer registry cencer’s data | Cohort  1992-2006 | 6,356/88(33 gynecologic cancer) | First cancer defined as women older than 20 years with histologically confirmed invasive breast cancer. | Breast cancer patients with at least 1 month of follow-up were considered.  SIRs reported for: endometrium, ovary, cervix uteri  IARC coding: yes | All: ovary, endometrial | High (++) |
| Mellemkjær1 2011 (16) | Cancer Registries of Denmark,  Norway,  Finland | Cohort  1943–2006  Follow-up  until 2006  (Denmark and  Finland), and  2007 (Norway) | 304,703/23,304 | Women older than 20 years, diagnosed with invasive first primary breast cancer were included. | All second cancers (except non-melanoma) diagnosed more than 1 month after the first breast cancer diagnosis were included.  SIRs reported for: Salivary glands, esophagus, lung, pleura, bone,  connective tissue, thyroid gland, leukemia, endometrial, ovarian,  mouth, pharynx, liver, larynx colorectum, gallbladder and bile  ducts, pancreas, kidney  IARC rules: yes | All: esophagus, lung, bone, connective tissue, thyroid, leukemia, .endometrium, ovary, liver, colon and rectum, pancreas, kidney, stomach, melanoma, lymphoma.  <40 y: esophagus, lung, bone, connective tissue, thyroid, leukemia, endometrium, ovary, colon and rectum, pancreas, kidney, stomach, eye.  40–49 y: esophagus, lung, connective tissue, leukemia, endometrium, ovary, stomach  50–59 y: esophagus, connective tissue, endometrium, ovary, colon and rectum, stomach  60–069 y: esophagus, connective tissue, endometrium, kidney, stomach  ≥70 y: endometrium | High (++) |
| Fallah 2011 (17) | Sweden/ Swedish Family-Cancer Database | Cohort  1958-2008 | -/272 | Women survived from first primary breast cancer. | MEN syndrome related parathyroid adenoma and patients with no identified first-degree relative were excluded.  Sir reported for: parathyroid adenoma.  IARC coding: not said, but Swedish Family-Cancer Database follow IARC rules. | All: parathyroid adenoma | Low (-)  Selection bias: patients with a history of MEN-related tumours in their first-degree relatives and patients with no identified first-degree relative were excluded. Also, the number of first primary breast cancer didn’t mention. |
| AIRTUM Working Group 2013 (18) | Italy/AIRTUM population-based cancer registries | Cohort  1976-2010 | 224,228/11,310 | All men and women whose first primary breast cancer occurred during the research period. | Second cancers defined as diagnosed cancer at ≥0 months (including synchronous and metachronous cancers) and ≥2 months (including metachronous cancers) after the first primary cancer.  SIRs reported for: head & neck (oral cavity, pharynx, larynx), esophagus, stomach, colorectum, liver, gallbladder, pancreas, lung, skin melanoma, mesothelioma, Kaposi sarcoma, soft tissue, bone, breast, prostate, testis, corpus uteri, cervix uteri, ovary, kidney and renal pelvis, bladder and urinary tract, brain and central nervous system, thyroid, Hodgkin and non-Hodgkin lymphoma, multiple myeloma, and leukemia.  IARC coding: yes | For men:  0≥months: all sites but skin and breast, testis, kidney and renal pelvis  ≥2 months: kidney and renal pelvis   - 1. months: all sites but skin and breast, testis, gallbladder   2-11 months: kidney and renal pelvis, thyroid  For women:  0≥months: all sites but skin and breast, esophagus, stomach, colorectum, liver gallbladder, skin melanoma, lung, soft tissue, bone, corpus uteri, ovary, kidney and renal pelvis, bladder and urinary tract, brain and central nervous system, thyroid, leukemia, other and ill-defined sites.  ≥2 months: all sites but skin and breast, esophagus, stomach, colorectum, liver gallbladder, skin melanoma, Kaposi sarcoma, soft tissue, breast, corpus uteri, ovary, kidney and renal pelvis, bladder and urinary tract, brain and central nervous system, thyroid, leukemia, and other and ill-defined sites.  0-1months: all sites but skin and breast, esophagus, lung, skin melanoma, breast, corpus uteri, ovary, kidney and renal pelvis, thyroid, Hodgkin and non-Hodgkin lymphoma, and other and ill-defined sites.  2-11 months: gallbladder, breast, kidney and renal pelvis, and other and ill-defined sites.  1-5y: all sites but skin and breast, colorectum, skin melanoma, breast, corpus uteri, ovary, brain and nervous system, thyroid, leukemia, and other and ill-defined sites.  5-10y: all sites but skin and breast, stomach, liver, pancreas, soft tissue, breast, corpus uteri, ovary, kidney and renal pelvis, bladder and urinary tract, brain and nervous system, thyroid, leukemia, and other and ill-defined sites.  ≥10y: all sites but skin and breast, esophagus, stomach, colorectum, soft tissue, breast, corpus uteri, ovary, and other and ill-defined sites. | High (++) |
| Molina-Montes 2013 (19) | Spain/Granada Cancer  Registry | Cohort  1985-2007 | 5,897/314 | Women diagnosed with an invasive first primary breast cancer (ICD-O3: C50). Women with synchronous tumors, and same day of death and diagnosis of breast cancer diagnosis were excluded. | Second cancers diagnosed after more than 3 months after the first breast cancer diagnosis.  SIRs reported for: Endometrium, colon, rectum, stomach, ovary, thyroid gland, skin non-melanoma, kidney, bladder, hematologic malignancies.  IARC rules: yes | All: endometrium, non-melanoma skin.  <50 y: ovary and skin non-melanoma.  ≥50 y: endometrium, skin non-melanoma | High (++) |
| Utada 2014 (20) | Japan/ Nagasaki Prefecture Cancer Registry | Cohort  1985-2007  Followed until 2008 | -/235 | Females with primary breast cancer (ICD9) were included. | Second tumors in the same site as the first primary cancer were excluded.  SIRs reported for: lung, corpus uteri, ovary thyroid.  IARC coding: yes | All: Lung, corpus uteri, ovary thyroid. | Acceptable (+)  Selection bias: the number of first primary breast cancer didn’t mention. |
| Levi 2014 (21) | Switzerland/Vaud  and Neuchaˆtel Swiss Registries | Cohort  1976-2010 | 17,329/34 | Women diagnosed with breast cancer as first cancer during 1976-2010. | Following ICD morphological codes for sarcomas were considered as second cancer: 8800–4, 8810–32, 8850–70, 8890–1, 8900–20, 8930, 8951, 8990, 9020, 9120, 9130,9140, 9180–4, 9220, 9240, 9260, 9370, 9522, 9560, 9580  SIR reported for: sarcoma.  IARC coding: yes | All: sarcoma  Age at diagnosis:  <70y:sarcoma  ≥70y: sarcoma  Time since first breast cancer:  ≥5y: sarcoma | High (++) |
| An 2015 (22) | South Korea/ a single tertiary referral canter, at  Seoul National University Hospital | Retrospective case-controlled  1970-2009 | 6,833/81 | Only infiltrative ductal carcinoma [IDC], ductal carcinoma in situ [DCIS], and others that women experienced for the first time in their life without a previous history of cancer and underwent curative surgery was considered as primary cancer. | Second primary cancer defined as diagnosed cancer at least two years after the first primary cancer.  SIR reported for: thyroid  IARC coding: not said, but South Korea follow the IARC rules. | All: Thyroid | Acceptable (+)  Selection bias: Due to including only women with breast cancer that underwent curative surgery as the first primary cancer. |
| Ricceri 2015 (23) | 23 centres from Denmark,  France, Germany, Greece, Italy, the Netherlands, Norway,  Spain, Sweden and the United Kingdom | Cohort  Follow-up duration: 11 years  The follow-up was based on population cancer registries,  except in France, Germany and Greece, where a combination of health insurance records, cancer and pathology registries, and active follow-up were used. | 10,045/352 | Population of this study defined as woman participate in The European Prospective Investigation into Cancer and Nutrition (EPIC) which developed primary breast cancer without history of prior cancer. | Non-melanoma skin cancers and synchronous tumours (i.e., same date of diagnosis) were excluded.  SIRs reported for: colorectum, pancreas, lung, melanoma, breast, endometrium, ovary, kidney, thyroid, lymphoma, all cancer, all but breast cancers.  IARC coding: yes | All: colorectal cancer, lymphoma, melanoma, endometrium, and kidney cancers | Acceptable (+)  Selection bias: Most of the patients were obtained from the general population, except the  French cohort (mostly teachers), the Utrecht and  the Florence cohorts (women screening for breast cancer), part of the other Italian and  Spanish cohorts (blood donors) and the Oxford cohort (mostly on vegetarians). |
| Hung 2016 (24) | Taiwan/Registry of Catastrophic Illness | Cohort  1997-2011 | 101,493/2,674 | males and females with primary breast cancer  (ICD-  9-CM, code 174–175) without history of prior cancer were included. | Patients who had at least 1 year of follow-up after breast cancer diagnosis and had complete personal information were included.  SIRs reported for: all cancers, esophagus, stomach, colon & rectum & anus, liver and biliary tract, pancreas, lung and mediastinum, bone and soft tissue, skin, cervix, uterus, ovary, prostate, bladder, kidney, thyroid, hematologic malignancies, all other.  IARC coding: not said, but Registry of Catastrophic Illness follow IARC rules. | All women: uterine, thyroid bone, soft tissue  All men: thyroid, skin, head & neck. | High (++) |
| Bazire 2017 (25) | France/ Curie institute, Paris. | Cohort  1981-2000 | 17,745/867 | Female breast cancer patients without previous cancer, bilateral breast cancer or metastatic breast cancer were considered as first primary cancer. | Second cancer defined as diagnosed cancer more than 1 year after breast cancer and patient should be followed-up at least two years.  SIRs reported for: esophagus, colorectum, others gastrointestinal, ovarian, others gynecological, leukemia, lymphoma, melanoma, head and neck, lung, sarcoma, thyroid, and genitourinary.  IARC coding: not said, but France follow the IARC rules. | All: Colorectum, ovarian, others gynecological, leukemia, lymphoma, head and neck, lung, sarcoma | High (++) |
| Silverman 2017 (26) | Israeli/ Israel National Cancer Registry | Cohort  1992-2006  Follow-up until 2011 | 46,090/3,980 | Women diagnosed with invasive breast cancer (excluding breast lymphomas) between 1990-2006, and without prior cancer diagnosis were included. | Subsequent non–breast cancer occurring at ≥0 and ≥6 months after breast cancer diagnosis were included. Patients with more than one cancer during the study period were excluded after the first cancer diagnosis.  SIRs reported for: all cancers, colorectal, uterus, lung, ovary, non-Hodgkin lymphoma, brain, invasive melanoma, thyroid, leukemia, uterine cervix.  IARC coding: yes | All: all cancers, colorectal, uterus, lung, ovary, invasive melanoma, thyroid, leukemia. | High (++) |
| Chen 2018 (27) | Taiwan/Taiwan Cancer Registry | Cohort  1979-2008 | 86,827/378 | Women older than 20 years who were diagnosed with breast cancer (ICD-9: 174). | Second cancer defined as cancer diagnosed more than 2 months after the primary cancer.  SIRs reported for: ovary and uterine cancers.  IARC coding: yes | All: uterine corpus, ovary  <50 years: uterine corpus, ovary  ≥50 y: uterine corpus, ovary  Time since diagnosis of first cancer:  ≤5y: uterine corpus, ovary  5-10y: uterine corpus, ovary  >10y: uterine corpus | Acceptable (+)  Selection bias: Exclusions applied  to women with unknown  information on missing birth dates, last follow-up date,  or death status. |
| Bright 2019 (28) | England and Wales/ the Office for National Statistics and the Welsh Cancer registry | Cohort  Jan 1 1971- Dec 31 2006  Follow-up end: Dec 31, 2012 | 36,236/1,877 | Women aged 15-39 years with malignant breast cancer as first cancer were included. | Patients who had not survived 5 years from their first cancer, second cancer occurred in contralateral paired organ and near first primary cancer site were excluded.  SIRs reported for: breast, cervix, Hodgkin lymphoma, melanoma, central nervous system, colorectal, non-Hodgkin lymphoma, thyroid, soft tissue sarcoma, ovary, bladder, female genital, leukemia, and head & neck.  IARC coding: yes | All: Lung and bronchus, clolorectal, melanoma, ovary, Corpus uteri, Other female  Genital, | Acceptable (+)  Selection bias: due to excluding patients that not survived from their first neoplasm. |
| Jabagi 2019 (29) | France/ French National Health Data System | Cohort  2006-2015  follow-up until 2016 | 439,704/3,046 | Females aged 20 to 85 years with invasive primary breast cancer (ICD-10, codes C50, D05) without history of prior cancer were included. | Second cancers defined as hematologic malignant neoplasm occurred at least 6 months after breast cancer diagnosis.  SIRs reported for: Acute myeloid leukemia, Myelodysplastic syndrome, Myeloproliferative  Neoplasm, Multiple myeloma, Hodgkin/non–Hodgkin  Lymphoma, Acute lymphoblastic leukemia/lymphocytic lymphoma.  IARC coding: not said, but French National Health Data System follow IARC rules. | All: Acute myeloid leukemia, Myelodysplastic syndrome, Multiple myeloma, Acute lymphoblastic leukemia/lymphocytic lymphoma | High (++) |
| Sundbøll 2020 (30) | Denmark/ Danish Cancer Registry | Cohort  1990-2017 | 84,972/2,020 | women older than 18 years with localized or regional spread breast tumors (according to  ICD-10) diagnosed from 1978 without history of cancer were included. | All urological and genital cancers after first breast cancer considered as second cancer.  SIRs reported for: kidney, urinary bladder, urological cancers, ovary, uterus, cervix, genital cancers  IARC coding: yes | All:  Age at diagnosis:  18-49y:bladder, ovary  50-59y:bladder, ovary, uterus  60-69y: uterus  ≥70y: uterus  Time since first primary cancer:  0-1y: Urological cancers, Kidney, Genital cancers, Ovary  2-5y: Urological cancers, Genital cancers, Uterus, Ovary  6-9y: kidney, genital cancers, uterus  ≥10y: urinary bladder, genital cancers, ovary | High (++) |

**References**

1. Prior P, Waterhouse JA. Multiple primary cancers of the breast and ovary. British journal of cancer. 1981;44(5):628-36.

2. Murakami R, Hiyama T, Hanai A, Fujimoto I. Second primary cancers following female breast cancer in Osaka, Japan--a population-based cohort study. Japanese journal of clinical oncology. 1987;17(4):293-302.

3. Brenner H, Siegle S, Stegmaier C, Ziegler H. Second primary neoplasms following breast cancer in Saarland, Germany, 1968-1987. European journal of cancer (Oxford, England : 1990). 1993;29a(10):1410-4.

4. Rubino C, de Vathaire F, Diallo I, Shamsaldin A, Lê MG. Increased risk of second cancers following breast cancer: role of the initial treatment. Breast cancer research and treatment. 2000;61(3):183-95.

5. Tanaka H, Tsukuma H, Koyama H, Kinoshita Y, Kinoshita N, Oshima A. Second primary cancers following breast cancer in the Japanese female population. Japanese journal of cancer research : Gann. 2001;92(1):1-8.

6. Evans HS, Lewis CM, Robinson D, Bell CM, Møller H, Hodgson SV. Incidence of multiple primary cancers in a cohort of women diagnosed with breast cancer in southeast England. British journal of cancer. 2001;84(3):435-40.

7. Levi F, Te VC, Randimbison L, La Vecchia C. Cancer risk in women with previous breast cancer. Annals of oncology : official journal of the European Society for Medical Oncology. 2003;14(1):71-3.

8. Sadetzki S, Calderon-Margalit R, Peretz C, Novikov I, Barchana M, Papa MZ. Second primary breast and thyroid cancers (Israel). Cancer Causes & Control. 2003;14(4):367-75.

9. Hemminki K, Scélo G, Boffetta P, Mellemkjaer L, Tracey E, Andersen A, et al. Second primary malignancies in patients with male breast cancer. British journal of cancer. 2005;92(7):1288-92.

10. Mellemkjær L, Friis S, Olsen JH, Scélo G, Hemminki K, Tracey E, et al. Risk of second cancer among women with breast cancer. International journal of cancer. 2006;118(9):2285-92.

11. Prochazka M, Hall P, Granath F, Czene K. Family history of breast cancer and young age at diagnosis of breast cancer increase risk of second primary malignancies in women: A population-based cohort study. British journal of cancer. 2006;95(9):1291-5.

12. Lee KD, Chen SC, Chan CH, Lu CH, Chen CG, Lin JT, et al. Increased risk for second primary malignancies in women with breast cancer diagnosed at young age: A population-based study in Taiwan. Cancer Epidemiology Biomarkers and Prevention. 2008;17(10):2647-55.

13. Schaapveld M, Visser O, Louwman MJ, de Vries EG, Willemse PH, Otter R, et al. Risk of new primary nonbreast cancers after breast cancer treatment: a Dutch population-based study. Journal of clinical oncology : official journal of the American Society of Clinical Oncology. 2008;26(8):1239-46.

14. Verkooijen HM, Fioretta G, Rapiti E, Vlastos G, Neyroud-Caspar I, Chappuis PO, et al. Family history of breast or ovarian cancer modifies the risk of secondary leukemia after breast cancer: results from a population-based study. International journal of cancer. 2008;122(5):1114-7.

15. Gulhan I, Eser S, Yakut C, Bige O, Ilhan E, Yildirim Y, et al. Second primary gynecologic cancers after breast cancer in Turkish women. International journal of gynecological cancer : official journal of the International Gynecological Cancer Society. 2009;19(4):648-50.

16. Mellemkjær L, Christensen J, Frederiksen K, Pukkala E, Weiderpass E, Bray F, et al. Risk of Primary Non–Breast Cancer After Female Breast Cancer by Age at DiagnosisCancer After Breast Cancer. Cancer epidemiology, biomarkers & prevention. 2011;20(8):1784-92.

17. Fallah M, Kharazmi E, Sundquist J, Hemminki K. Nonendocrine cancers associated with benign and malignant parathyroid tumors. The Journal of clinical endocrinology and metabolism. 2011;96(7):E1108-14.

18. Group AW. Italian cancer figures, report 2013: Multiple tumours. Epidemiologia e prevenzione. 2013;37(4-5 Suppl 1):1-152.

19. Molina-Montes E, Pollán M, Payer T, Molina E, Dávila-Arias C, Sánchez MJ. Risk of second primary cancer among women with breast cancer: A population-based study in Granada (Spain). Gynecologic oncology. 2013;130(2):340-5.

20. Utada M, Ohno Y, Hori M, Soda M. Incidence of multiple primary cancers and interval between first and second primary cancers. Cancer Science. 2014;105(7):890-6.

21. Levi F, Randimbison L, Maspoli-Conconi M, Blanc-Moya R, La Vecchia C. Incidence of second sarcomas: a cancer registry-based study. Cancer Causes & Control. 2014;25(4):473-7.

22. An JH, Hwangbo Y, Ahn HY, Keam B, Lee KE, Han W, et al. A Possible Association Between Thyroid Cancer and Breast Cancer. Thyroid : official journal of the American Thyroid Association. 2015;25(12):1330-8.

23. Ricceri F, Fasanelli F, Giraudo MT, Sieri S, Tumino R, Mattiello A, et al. Risk of second primary malignancies in women with breast cancer: Results from the European prospective investigation into cancer and nutrition (EPIC). International journal of cancer. 2015;137(4):940-8.

24. Hung MH, Liu CJ, Teng CJ, Hu YW, Yeh CM, Chen SC, et al. Risk of Second Non-Breast Primary Cancer in Male and Female Breast Cancer Patients: A Population-Based Cohort Study. PloS one. 2016;11(2):e0148597.

25. Bazire L, De Rycke Y, Asselain B, Fourquet A, Kirova YM. Risks of second malignancies after breast cancer treatment: Long-term results. Cancer radiotherapie : journal de la Societe francaise de radiotherapie oncologique. 2017;21(1):10-5.

26. Silverman BG, Lipshitz I, Keinan-Boker L. Second primary cancers after primary breast cancer diagnosis in Israeli women, 1992 to 2006. Journal of global oncology. 2017;3(2):135-42.

27. Chen MC, Lee KD, Lu CH, Wang TY, Huang SH, Chen CY. The bidirectional association among female hormone-related cancers: breast, ovary, and uterine corpus. Cancer medicine. 2018;7(6):2299-306.

28. Bright CJ, Reulen RC, Winter DL, Stark DP, McCabe MG, Edgar AB, et al. Risk of subsequent primary neoplasms in survivors of adolescent and young adult cancer (Teenage and Young Adult Cancer Survivor Study): a population-based, cohort study. The Lancet Oncology. 2019;20(4):531-45.

29. Jabagi MJ, Vey N, Goncalves A, Le Tri T, Zureik M, Dray-Spira R. Evaluation of the Incidence of Hematologic Malignant Neoplasms Among Breast Cancer Survivors in France. JAMA network open. 2019;2(1):e187147.

30. Sundboll J, Farkas DK, Adelborg K, Schapira L, Tamang S, Norgaard M, et al. Risk of primary urological and genital cancers following incident breast cancer: a Danish population-based cohort study. Breast cancer research and treatment. 2020;184(3):825-37.

Supplementary Table 2 comprehensive search strategy (7 October 2022)

| **Database** | **Search by** | **Search items** |
| --- | --- | --- |
| PubMed | MeSH terms | 1. (((("Neoplasms, Multiple Primary/epidemiology"[Mesh]) OR "Neoplasms, Multiple Primary/prevention and control"[Mesh]) OR "Neoplasms, Second Primary/epidemiology"[Mesh]) OR "Neoplasms, Second Primary/prevention and control"[Mesh]) AND "Breast Neoplasms/epidemiology"[Mesh] N=916 2. Limits: Humans, English, and Adult: 19+ years. N=657 |
|  | Keywords | 1. Breast cancer N=475,181 2. Second cancer N=172,046 3. Second malignancies N=48,813 4. Multiple primary cancer N=66,846 5. Multiple primary malignancies N=61,604 6. #2 OR #3 OR #4 OR #5 N=235,361 7. #1 AND #6 N=31,219 8. Risk N=3,132,162 9. Population-based N=156,699 10. #7 AND #8 AND #9 N=627 11. Limits: Humans, English, and Adult: 19+ years. N=462 |
| Scopus | Keywords | 1. Limits: ( ( ( TITLE-ABS-KEY ( second AND cancer ) OR TITLE-ABS-KEY ( second AND malignancies ) OR TITLE-ABS-KEY ( multiple AND primary AND cancer ) OR TITLE-ABS-KEY ( multiple AND primary AND malignancies ) ) ) AND ( TITLE-ABS-KEY ( breast AND cancer ) ) ) AND ( TITLE-ABS-KEY ( population-based ) ) AND ( TITLE-ABS-KEY ( risk ) ) AND ( EXCLUDE ( DOCTYPE , "cp" ) OR EXCLUDE ( DOCTYPE , "le" ) OR EXCLUDE ( DOCTYPE , "ch" ) OR EXCLUDE ( DOCTYPE , "sh" ) ) AND ( EXCLUDE ( SRCTYPE , "k" ) OR EXCLUDE ( SRCTYPE , "b" ) ) AND ( LIMIT-TO ( LANGUAGE , "English" ) ) AND ( EXCLUDE ( DOCTYPE , "re" ) ) N=585 |
| Web of Science | Keywords | 1. (((TS=(second cancer)) OR TS=(multiple primary malignancies)) OR TS=(multiple primary cancer)) OR TS=(second malignancies) N=133,620 2. TS=(breast cancer) N=646,261 3. (TS=(risk)) OR TS=(incidence) N=4,605,844 4. TS=(population-based) N=184,815 5. #1 AND #2 AND #3 AND #4 N=711 6. Limit: English N=703 |
| Cochrane library | Keywords | 1. Breast cancer N=41,090 2. (risk):ti,ab,kw OR (incidence):ti,ab,kw N=357,922 3. MeSH descriptor: [Neoplasms, Second Primary] explode all trees N=484 4. MeSH descriptor: [Neoplasms, Multiple Primary] explode all trees N=170 5. #3 OR #4 N=654 6. #1 AND #2 AND #5 N=64 |

Supplementary Table 3 Methodological quality assessment using the SIGN50 Scottish Intercollegiate Network checklist

| **First author (year)** | **Selection of subjects** | **Assessment** | **Confounding** | **Statistical analysis** | **Overall quality** |
| --- | --- | --- | --- | --- | --- |
| Prior (1981) | ✓ | ✓ | ✓ | ✓ | High ++ |
| Murakami (1987) | ✘ | ✓ | ✓ | ✓ | Acceptable + |
| Brenner (1993) | ✓ | ✓ | ✓ | ✓ | High ++ |
| Rubino (2000) | ✘ | ✓ | ✘ | ✓ | Low - |
| Evans (2001) | ✘ | ✓ | ✓ | ✓ | Acceptable + |
| Tanaka (2001) | ✓ | ✓ | ✓ | ✓ | High ++ |
| Levi (2003) | ✓ | ✓ | ✓ | ✓ | High ++ |
| Sadetzki (2003) | ✓ | ✓ | ✓ | ✓ | High ++ |
| Hemminki (2005) | ✓ | ✓ | ✓ | ✓ | High ++ |
| Mellemkjær (2006) | ✓ | ✓ | ✓ | ✓ | High ++ |
| Prochazka (2006) | ✓ | ✓ | ✓ | ✓ | High ++ |
| Lee (2008) | ✓ | ✓ | ✓ | ✓ | High ++ |
| Schaapveld (2008) | ✓ | ✓ | ✘ | ✓ | Acceptable - |
| Verkooijen (2008) | ✓ | ✓ | ✓ | ✓ | High ++ |
| Gulhan (2009) | ✓ | ✓ | ✓ | ✓ | High ++ |
| Fallah (2011) | ✘ | ✓ | ✓ | ✓ | Acceptable + |
| Mellemkjær (2011) | ✓ | ✓ | ✓ | ✓ | High ++ |
| AIRTUM Working Group (2013) | ✓ | ✓ | ✓ | ✓ | High ++ |
| Molina-Montes (2013) | ✓ | ✓ | ✓ | ✓ | High ++ |
| Levi (2014) | ✓ | ✓ | ✓ | ✓ | High ++ |
| Utada (2014) | ✘ | ✓ | ✓ | ✓ | Acceptable + |
| An (2015) | ✘ | ✓ | ✓ | ✓ | Acceptable + |
| Ricceri (2015) | ✘ | ✓ | ✓ | ✓ | Acceptable + |
| Hung (2016) | ✓ | ✓ | ✓ | ✓ | High ++ |
| Bazire (2017) | ✓ | ✓ | ✓ | ✓ | High ++ |
| Silverman (2017) | ✓ | ✓ | ✓ | ✓ | High ++ |
| Chen (2018) | ✘ | ✓ | ✓ | ✓ | Acceptable + |
| Bright (2019) | ✘ | ✓ | ✓ | ✓ | Acceptable + |
| Jabagi (2019) | ✓ | ✓ | ✓ | ✓ | High ++ |
| Sundboll (2020) | ✓ | ✓ | ✓ | ✓ | High ++ |
